# Efficacy of Prehabilitation Including Exercise on Postoperative Outcomes Following Abdominal Cancer Surgery: A Systematic Review and Meta-Analysis

**DOI:** 10.3389/fsurg.2021.628848

**Published:** 2021-03-19

**Authors:** Jamie L. Waterland, Orla McCourt, Lara Edbrooke, Catherine L. Granger, Hilmy Ismail, Bernhard Riedel, Linda Denehy

**Affiliations:** ^1^Department of Anaesthesia, Perioperative and Pain Medicine, Peter MacCallum Cancer Centre, Melbourne, VIC, Australia; ^2^Department of Physiotherapy, The University of Melbourne, Melbourne, VIC, Australia; ^3^Division of Allied Health, Peter MacCallum Cancer Centre, Melbourne, VIC, Australia; ^4^Cancer Institute, University College London, London, United Kingdom; ^5^Physiotherapy Department, The Royal Melbourne Hospital, Melbourne, VIC, Australia; ^6^Centre for Integrated Critical Care, The University of Melbourne, Melbourne, VIC, Australia

**Keywords:** prehabilitation, cancer, systematic review, surgery, meta-analysis

## Abstract

**Objectives:** This systematic review set out to identify, evaluate and synthesise the evidence examining the effect of prehabilitation including exercise on postoperative outcomes following abdominal cancer surgery.

**Methods:** Five electronic databases (MEDLINE 1946-2020, EMBASE 1947-2020, CINAHL 1937-2020, PEDro 1999-2020, and Cochrane Central Registry of Controlled Trials 1991-2020) were systematically searched (until August 2020) for randomised controlled trials (RCTs) that investigated the effects of prehabilitation interventions in patients undergoing abdominal cancer surgery. This review included any form of prehabilitation either unimodal or multimodal that included whole body and/or respiratory exercises as a stand-alone intervention or in addition to other prehabilitation interventions (such as nutrition and psychology) compared to standard care.

**Results:** Twenty-two studies were included in the systematic review and 21 studies in the meta-analysis. There was moderate quality of evidence that multimodal prehabilitation improves pre-operative functional capacity as measured by 6 min walk distance (Mean difference [MD] 33.09 metres, 95% CI 17.69–48.50; *p* = <0.01) but improvement in cardiorespiratory fitness such as preoperative oxygen consumption at peak exercise (VO_2_ peak; MD 1.74 mL/kg/min, 95% CI −0.03–3.50; *p* = 0.05) and anaerobic threshold (AT; MD 1.21 mL/kg/min, 95% CI −0.34–2.76; *p* = 0.13) were not significant. A reduction in hospital length of stay (MD 3.68 days, 95% CI 0.92–6.44; *p* = 0.009) was observed but no effect was observed for postoperative complications (Odds Ratio [OR] 0.81, 95% CI 0.55–1.18; *p* = 0.27), pulmonary complications (OR 0.53, 95% CI 0.28–1.01; *p* = 0.05), hospital re-admission (OR 1.07, 95% CI 0.61–1.90; *p* = 0.81) or postoperative mortality (OR 0.95, 95% CI 0.43–2.09, *p* = 0.90).

**Conclusion:** Multimodal prehabilitation improves preoperative functional capacity with reduction in hospital length of stay. This supports the need for ongoing research on innovative cost-effective prehabilitation approaches, research within large multicentre studies to verify this effect and to explore implementation strategies within clinical practise.

## Introduction

Healthcare is under increasing pressure to ensure that perioperative care is patient-centred and value-based (1–4). “Prehabilitation” aims to optimise physiological reserve and address modifiable risk factors prior to surgery to improve postoperative outcomes (2). In cancer care, prehabilitation is a process on the continuum of care that occurs between cancer diagnosis and the beginning of acute treatment (usually surgery) (5) and includes interventions that promote physical and psychological health to reduce the incidence and/or severity of future impairments. Previously, prehabilitation programs focused solely on unimodal exercise interventions however recently there has been a growing evidence-base supporting multimodal prehabilitation including respiratory, aerobic and/or resistance training programs as well as nutritional and psychological interventions (6).

There are conflicting results regarding the effectiveness of prehabilitation in patients with cancer awaiting surgery (7, 8). Similarly, the optimal approach to delivering prehabilitation is unknown with programs differing in terms of exercise type, training frequency, intensity, duration and supervision, and thus therapeutic validity (7). While multimodal programs may intuitively be the best way to support patients with cancer there is limited evidence supporting superiority of multimodal vs. unimodal interventions (6).

Although individual programs have been shown to increase preoperative fitness (9), heterogeneity in study designs has limited the synthesis of evidence regarding effects on postoperative outcomes in those undergoing abdominal surgery for cancer (6, 7). Several randomised controlled trials (RCTs) (8, 10–15) have been published since the last systematic review in this field of research (7). This systematic review set out to evaluate and synthesise the evidence examining the effect of prehabilitation on postoperative outcomes in patients undergoing abdominal cancer surgery.

## Methods

This systematic review was conducted in accordance with the Cochrane Collaboration methods (16), reported according to the Preferred Reporting Items for Systematic Reviews and Meta-analysis (PRISMA) checklist (17) and registered with the International Prospective Register of Systematic Reviews (PROSPERO 2020 CRD42020166551).

### Study Selection

RCTs and pseudo-randomised controlled trials (such as those that allocate participants to groups based on location of residence or date of assessment) of prehabilitation, including whole body or respiratory exercise, for adults (18 years) preparing for major abdominal cancer surgery that were published in English between January 2010 and August 2020 and met the inclusion criteria (Table 1) were identified by using our predefined search criteria (Supplementary Material 1) within the following databases: Ovid MEDLINE, Embase Classic+Embase, CINAHL Complete, Cochrane Central Register of Controlled Trials and PEDro (Physiotherapy Evidence database). Given that prehabilitation is a rapidly evolving field of research we restricted our search to publications within the last 10 years (January 1st, 2010 onwards). Reference lists of identified studies were reviewed for additional references. An additional rerun of the search criteria was conducted in August 2020 for any recently published studies.

**Table 1:** Inclusion criteria.

**Criteria**	**Category**	**Description**
Inclusion criteria	Design	• RCTs or pseudo RCTs
	Participants	• Adults 18 years scheduled to undergo abdominal surgery for cancer with at least 10 study participants.
	Intervention	• Studies that evaluated a modality of exercise prehabilitation, including whole body or respiratory exercises, including education as a stand-alone intervention or included with a framework of multimodal interventions
	Comparison	• A similar patient-group that was not exposed to a prehabilitation program (e.g., standard care with no intervention).
	Outcome measures	• Studies that include a measure of cardiorespiratory fitness/functional capacity and/or measures of postoperative outcome

*RCTs, randomised controlled trials*.

Search results were imported into the Covidence systematic literature review software program (https://www.covidence.org; Veritas Health Innovation Ltd, Australia) (18). Two of the review authors (JW, OM) independently screened the identified studies based on their title and abstract. When there was insufficient information to determine eligibility, full texts were retrieved and screened. A third researcher (LD, LE) was available for discussion could a consensus not be reached between the two reviewers on study inclusion.

### Data Extraction

Two of the review authors (JW, OM) independently extracted data from the included studies using a standardised form. The clinical and outcome data extracted included the patient's baseline characteristics, baseline cardiorespiratory fitness, functional capacity after prehabilitation, postoperative complications, ICU usage, hospital length of stay, hospital re-admission and postoperative mortality. Data were entered into Review Manager 5.4 to examine appropriateness for meta-analysis (19).

Prehabilitation program data were also extracted. These included program timeframes, components of multimodal interventions and details of the exercise intervention according to the consensus exercise reporting template (CERT) (20). The CERT is a 16-item checklist developed by an international panel of exercise experts that contains seven categories: materials, provider, delivery, location, dosage, tailoring and compliance. The CERT (Supplementary Material 1) describes exercise interventions and assists with the evaluation and understanding of exercise parameters (20).

### Data Synthesis and Analysis

Data were extracted from the included studies, pooled and analysed using random effects models after consideration of heterogeneity between the various studies. For continuous outcomes, data were calculated as mean differences (MD) when data were on a uniform scale and standardised mean differences (SMD) with 95% confidence intervals (95% CI) when data were presented using different scales. The estimated effect size was calculated for outcomes reported in three or more studies. For dichotomous variables, individual and pooled statistics were calculated as odds ratios (OR) with 95% CI. The 95% prediction interval (95% PI; Supplementary Material 1), an index that describes the true effect size for 95% of all comparable studies was used to assess heterogeneity (21). PIs were used instead of the inconsistency index (*I*^2^), which has been shown to over or under-estimate the true effect size across studies due to sampling error (21). PIs were calculated using an excel spreadsheet developed by Dr. Michael Borenstein, available at https://meta-analysis-books.com/. A *p* < 0.05 was considered to indicate statistical significance.

For continuous outcomes differences of means and variance of difference of means were obtained directly from the study results or calculated from the mean, variance and statistical significance on pre- and post- intervention assessments using RevMan meta-analysis software package (19) and the downloadable RevMan calculator available from Cochrane training (https://training.cochrane.org/resource/revman-calculator). Where the mean and SD of the change from baseline to endpoint were not reported in the original articles, the following equations were used to calculate them (16).

$$\begin{array}{l} Mean_{change}=Mean_{endpoint}-\;Mean_{baseline}\\ SD_{change}=\sqrt{{\left(SD_{baseline}\right)}^{2}+{\left(SD_{endpoint}\right)}^{2}-2\times r\times SD_{baseline}\times SD_{endpoint}} \end{array}$$

where *r* represents the correlation coefficient. We took *r* = 0.4 as a conservative estimate in this study (22).

Where data aggregation was not possible, due to clinical, methodological, or statistical heterogeneity, these results were summarised narratively.

Quality of evidence was analysed using the Grades of Research, Assessment, Development and Evaluation (GRADE) approach, which measures studies on six domains; study design grade, risk of bias, heterogeneity or inconsistency of effect, imprecision and publication bias to calculate a final grade (23). Data were independently appraised for the risk of bias of the included studies using version 2 of the Cochrane risk-of-bias tool for randomised trials (24).

## Results

**Figure 1:**
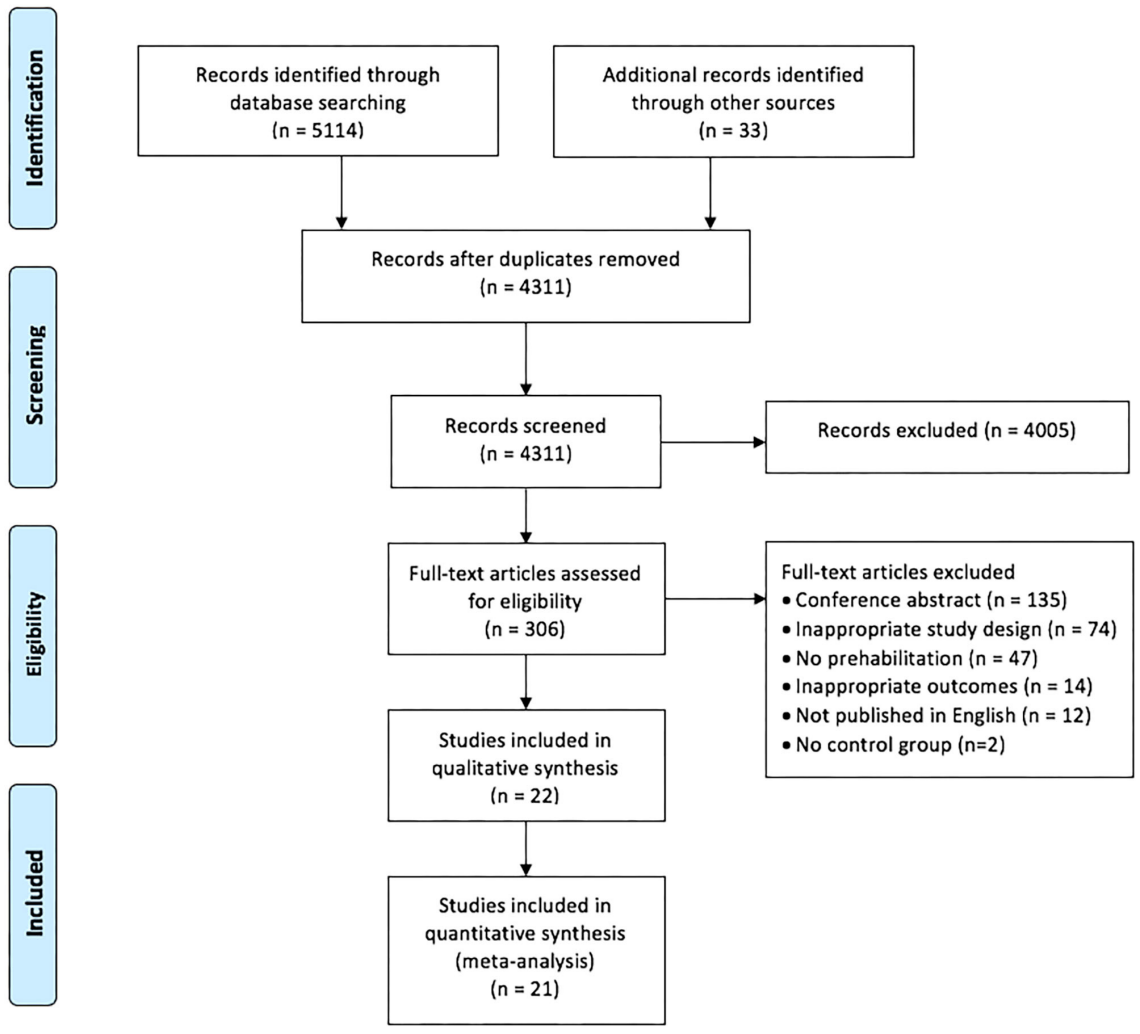
PRISMA flow chart of included and excluded studies within this systematic review and meta-analysis (25).

The search strategy for RCTs published between January 2010 and August 2020 yielded a total of 5,147 studies, and 4,311 studies after the exclusion of duplicates. Of these, 4,005 studies were excluded based on screening the title and abstract, leaving 306 full-text articles that were assessed for eligibility. Of these 284 studies were excluded: 135 were conference abstracts, 74 were not RCTs or pseudo-RCTs, 61 did not meet our review criteria for interventions and/or outcomes, two studies did not have a comparative usual care group and 12 studies were published in a language other than English (Figure 1 - PRISMA flow chart). Agreement between the two independent reviewers on title/abstracts and full text criteria was 91 and 96%, respectively, and two studies were referred to a third reviewer (LD) for final decision.

Meta-analysis was limited by methodological, clinical and statistical heterogeneity within the included studies. Additional data were requested for four of the studies (8, 26–28) with two able to provide the data requested (8, 27). Therefore, data was interpreted from a study figure (28), or calculated from other data within the study (26) for meta-analysis. For pooled data summary please see Supplementary Table 11.

### Study Characteristics

In total, this review included 22 studies, of which 21 were RCTs and one a pseudo-RCT. The majority of the studies were conducted in Canada [five studies (8, 13, 26, 29, 30)] and the UK [five studies (14, 15, 28, 31, 32)]. Two were international multicentre RCTs, conducted in Australasia (27) and Europe (33). Table 2 summarises the characteristics of studies included in the qualitative synthesis. A total of 1,700 participants were included in these studies, with sample sizes ranging from 21 to 296 patients and median ages ranging from 55 to 84 years of age across individual studies. Three studies included a variety of abdominal surgeries (27, 34, 35), seven studies focused on colorectal cancer (8, 12, 14, 15, 26, 29, 36), five on gastro-oesophageal cancer (11, 30, 33, 37, 38), four on urological cancer (13, 28, 31, 39), and single studies focused on pancreatic (10) and liver (40) cancers and one study on liver resection for colorectal metastatic disease (32).

**Table 2:** Description of included randomised controlled trials.

**References**	**Country**	**Population/pathology**	**Age (yr) (mean** **±** **SD, median (IQR))**		**Sample size, n (Male%)**	
			**Intervention**	**Control**	**Intervention**	**Control**
Blackwell et al. (28)	UK	Urological cancer	71 ± 2	72 ± 4	19 (100)	21 (95)
Carli et al. (8)	Canada	Colorectal cancer	78 (72–82)	82 (75–84)	55 (53)	55 (42)
Swaminathan et al. (37)	India	Gastric cancer	56.03 ± 14.95	56.82 ± 11.27	29 (62)	29 (69)
Ausania et al. (10)	Spain	Pancreatic cancer	65.9 (38–81)^‡^		18 (50)	22 (59)
Christensen et al. (11)^†^	Denmark	Gastro-oesophageal cancer	63.9 ± 8.2	65.5 ± 7.3	21 (86)	29 (93)
Karlsson et al. (12)^*^	Sweden	Colorectal cancer	83.5 (76–85)	74.0 (73–76)	10 (40)	11 (36)
Minnella et al. (13)	Canada	Bladder cancer	69.7 ± 10.2	66.0 ± 10.2	35 (63)	35 (77)
Moug et al. (14)	UK	Rectal cancer	65.2 ± 11.4	66.5 ± 9.6	24 (75)	24 (54)
Northgraves et al. (15)^*^	UK	Colorectal surgery	64.1 ± 10.5	63.5 ± 12.5	10 (40)	11 (64)
Banerjee et al. (31)^*^	UK	Bladder cancer	71.6 ± 6.8	72.5 ± 8.4	30 (90)	30 (87)
Barberan-Garcia et al. (34)	Spain	Elective major abdominal surgery	71 ± 11	71 ± 10	62 (68)	63 (80)
Boden et al. (27)	International^§^	Upper abdominal cancer	64 ± 13.0	69 ± 11.9	148	148
Bousquet-Dion et al. (29)	Canada	Colorectal cancer	74 (67.5–78)	71 (54.5–74.5)	37 (81)	26 (62)
Minnella et al. (30)	Canada	Esophagogastric cancer	67.3 ± 7.4	68.0 ± 11.6	26 (69)	25 (80)
Valkenet et al. (33)	International^§^	Oesophageal cancer	63.7 ± 7.5	62.7 ± 8.9	120 (74)	121 (80)
Dunne et al. (32)	UK	Colorectal liver metastasis	61 (56–66)	62 (53–72)	20 (65)	17 (77)
Jensen et al. (39)	Denmark	Bladder cancer	69 (66–72)^||^	71 (68–73)^||^	50 (78)	57 (70)
Yamana et al. (38)	Japan	Oesophageal cancer	68.33 ± 7.64	65.90 ± 9.50	30 (80)	30 (77)
Gillis et al. (26)	Canada	Colorectal cancer	65.7 ± 13.6	66.0 ± 9.1	38 (55)	39 (69)
Kaibori et al. (40)	Japan	Hepatocellular carcinoma	68.0 ± 9.1	71.3 ± 8.8	25 (68)	26 (73)
Soares et al. (35)	Brazil	Upper abdominal cancer	58.5 (51.3–63.5)	55.0 (49.3–64.3)	16 (50)	16 (56)
Dronkers et al. (36)	Netherlands	Colon cancer patients aged >60 years	71.1 ± 6.3	68.8 ± 6.4	22 (68)	20 (80)

* Feasibility Randomised Controlled Trial.

† Pseudo-randomised controlled trial.

‡ median (range), group ages not reported.

§ International: Valkenet 2018: Netherlands, Belgium, Ireland and Finland, Boden 2018: Australian and New Zealand.

|| *mean (95%CI)*.

#### Outcome Measures

Primary outcomes varied across the included studies, focusing on improving preoperative cardiorespiratory fitness (11, 28, 32), functional capacity (13, 26, 29, 30), and pulmonary function (35). Cardiorespiratory fitness was measured using Cardiopulmonary Exercise Test (CPET) variables (11, 31, 32, 34, 40) and estimated in one study using epidemiological data (36).The most common measure of functional capacity was the 6-min walk test (6MWT) (8, 12–15, 26, 29, 30, 34). Functional capacity was also measured using the 10-metre walk test (10MWT) (10), timed up and go (TUG) test (15, 36), and stair climb test (SCT) (15). Lower limb strength was measured using the 30-s sit-to-stand test (30STS) (12, 14) and chair rise time (CRT) (36).

The primary postoperative outcomes assessed included: postoperative complications (8, 10, 27, 33, 34, 38), and hospital length of stay (37, 39). Postoperative complications were measured using several different outcome measures, the Utrect Pneumonia Scoring System (38), Melbourne Group Score (27), Comprehensive Complications Index (8, 30) and the revised Uniform Pneumonia Score (33). The Clavien-Dindo rating scale was used to rate the severity of complications in the majority of included studies (8, 10–14, 26, 28–32, 34, 38, 39) (Supplementary Table 1). Some studies evaluated feasibility of the prehabilitation intervention (12, 14, 15, 31, 36) including the occurrence of serious adverse events that prevented surgery (11) or non-specific morbidity after surgery (40).

#### Exercise Interventions

##### Type

The type, frequency and intensity of the prehabilitation programs varied considerably across included studies. The majority of studies included multimodal interventions (8, 10, 12, 13, 26, 29, 30, 34–36, 38–40). Unimodal interventions included exercise interventions (11, 14, 15, 28, 31, 32), breathing exercise education (27), inspiratory muscle training (33), and incentive spirometry (37). Eight included studies combined other prehabilitation interventions with exercise: including nutrition interventions (8, 10, 13, 26, 29, 30, 39, 40), respiratory exercises (10, 35, 38), IMT (12, 35, 36) and psychological interventions (8, 13, 26, 29, 34) (Table 3). Table 3 summarises the prehabilitation components and Table 4 and Supplementary Tables 2–8 detail the exercise interventions according to the consensus reporting template (CERT) domains (20).

**Table 3:** Multimodal prehabilitation component.

	**Exercise**			**Respiratory**		**Nutrition**	**Psychology**	**Education session**
	**Aerobic**	**Resistance**	**Stretching**	**Exercises**	**IMT**			
**Multimodal**								
Carli et al. (8)	✓	✓				✓	✓	✓
Ausania et al. (10)	✓	✓		✓		✓		
Karlsson et al. (12)	✓	✓			✓			
Minnella et al. (13)	✓	✓				✓	✓	
Barbaren-Garcia et al. (34)	✓^*^	✓					✓	
Bousquet-Dion et al. (29)	✓	✓	✓			✓	✓	
Minnella et al. (30)	✓	✓				✓		
Jensen et al. (39)	✓	✓				✓		✓
Yamana et al. (38)	✓	✓		✓				✓
Gillis et al. (26)	✓	✓				✓	✓	
Kaibori et al. (40)	✓		✓			✓		
Soares et al. (35)	✓	✓	✓	✓	✓			
Dronkers et al. (36)	✓	✓			✓			
**Unimodal**								
Blackwell et al. (28)	✓^*^							
Swaminathan et al. (37)				**✓**^**†**^				
Christensen et al. (11)	✓^*^	✓						
Moug et al. (14)	✓							
Northgraves et al. (15)	✓	✓						
Banerjee et al. (31)	✓^*^							
Boden et al. (27)								✓
Valkenet et al. (33)					✓			
Dunne et al. (32)	✓^*^							

* High-intensity interval training.

† *Incentive spirometry. Further details on Exercise Component can be found in Table 4. IMT, Inspiratory Muscle Training*.

**Table 4:** Description of exercise prehabilitation intervention arms according to consensus exercise reporting template (CERT) domains (20) for three of the RCTs that included multimodal prehabilitation interventions as an example.

**CERT domain**	**Item no. and abbreviated item description**	**2020 Carli (8)**	**2018 Barberan-Garcia (34)**	**2018 Minnella (30)**
What	1. Type of exercise equipment	Recumbent stepper Resistance bands	Cycle-ergometer stationary bicycle	Resistance bands
Who	2. Qualifications, teaching/supervising expertise and/or training of the exercise instructor	Kinesiologist	Specialised Physiotherapist	Physician prescribed; Kinesiologist demonstrated
How	3. Whether exercise are performed individually or in a group	Not specified	Individually	Individual
	4. Whether exercises are supervised or unsupervised	Supervised and home based	Supervised	Unsupervised
	5. Measurement and reporting of adherence to exercise	Attendance at the in-hospital exercise session. Self-reported in diary and weekly telephone calls	Attendance at exercise sessions	Self-reported logbookWeekly telephone calls with kinesiologist
	6. Details of motivation strategies	CD with audio instructions. Weekly telephone calls	Motivational Interviewing and objective setting prior to exercise program and revisited throughout program	Weekly telephone calls with kinesiologist
	7. Decision rules for progressing the exercise program	No details in paper—references Bousquet-Dion 2018 for reporting of intervention	Peak work rate increased by ~5% every week up to a maximum of 85% peak work rate and 50% peak work rate for active rest.	Not reported
	8. Each exercise is described so that it can be replicated (e.g., illustrations, photographs)	No details in paper—references Bousquet-Dion 2018 for reporting of intervention	Detailed description provided	Aerobic described in terms of time, type, intensity (RPE), resistance less described
	9. Content of any home program component	Personalised progression of mod aerobic−30 min walking and resistance training x3/week	Personalised walking program focusing on increasing steps per day (using pedometer) and optimisation of walking intensity (using BORG scale)	All home based
	10. Non exercised components	Nutrition intervention +/– protein supplementation, psychology assessment, and personalised coping strategies, counselling for smoking and alcohol cessation.	Motivational interview	Nutrition assessment and supplements as needed.
	11. How adverse events that occur during exercise are documented and managed	No adverse events	No adverse events reported	No adverse events reported
Where	12. Setting in which exercises are performed	Hospital prehabilitation unit and home based	Community	Home based
When, how much	13. Detailed description of the exercises (e.g., set, reps, ration, intensity)	1 supervised session per week for 4 weeks. Warm up: 5 minAerobic: 30 min moderate intensity Resistance: 25 min, Stretching: 5 min	1–3 sessions per week Duration: 47 min Warm up: 5 min 30% peak work rate Intervals: 2 min 70%peak work rate, 3 min active rest 40% peak work rate Cool Down: 5 min 20% peak work rate Cycling Rate: 60-70RPM	Aerobic-−3 per week of 30 min moderate continuous training (incl 5 min warm up, 5 min cool down)BORG 12–13Strengthening 1 per week of 30 min (incl 5 min flexibility and 5 min stretching)–−3 sets x8-12 reps of 8 muscle groups.
Tailoring	14. Whether exercises are generic (“one size fits all”) or tailored to the individual	Personalised	Patient specific program	Individualised
	15. Decision rule that determines the starting level of exercise	No detail in paper—references Bousquet-Dion 2018 for reporting intervention.	Cardiopulmonary Exercise Test	Based on personal level and attitude. Based on BORG or 10 point resistance intensity scale.
How well	15. Whether the exercise intervention is delivered and performed as planned	Attendance of hospital sessions—mean (SD) 68% (38). Overall adherence 80% (27)	Nil reported	Overall compliance with programme reported (63%)

CERT tables for all included studies can be found in Supplementary 1.

*RPM, revolutions per minute; HR-max, maximum heart rate*.

##### Equipment

Eight (35%) of the studies used a cycle-ergometer (10, 11, 15, 28, 31, 32, 34, 38) for their exercise intervention and five (22%) used inspiratory muscle training (IMT) devices (12, 33, 35–37) to deliver breathing exercise interventions.

##### Exercise Program Detail

Only five studies provided criteria on when to progress exercise programs based on time in the program (14, 15, 28, 34, 35), determined by the instructor (11), using rate of perceived exertion (RPE) scales (12, 26, 29, 33, 36), percentage of maximum heart rate (31) or left to the participant to self-determine the progression (39). However, eight of these studies did not provide enough detail in the paper (8, 10, 13, 30, 32, 37, 38, 40) to enable replication. Exercise programs were described in detail to allow replication in a subset of studies (8, 12–14, 28, 31, 34), with aerobic exercise described in more detail than resistance exercises (10, 26, 29, 30, 38). Only one of the included studies provided a detailed supplementary file using pictures of exercise and equipment to allow replication (11, 15). Exercise programs were general or not reported in two studies (39, 40).

##### Motivational Strategies

Motivational strategies included within the exercise interventions were motivational interviewing (34), relaxation exercises (8), weekly telephone calls (8, 13, 26, 29, 30), instructional booklets (26, 29), instructional videos (33), discussion of mutual expectations and motivation (39), as well as information, motivation and encouragement provided during the session (15). One study employed behaviour change theory, providing a diary with targets and motivational material as well as engaging a support person to assist (14).

##### Supervision/Adherence

Twelve (55%) studies included some element of supervised intervention and ranged from one session (33) followed by a home program and up to three 60 min sessions per week (32, 34, 36, 40). Attendance at supervised exercise sessions measured adherence to treatment in six studies (8, 11, 28, 29, 31, 32, 34, 36), with one study also recording interruptions to attended sessions (15). Other studies monitored adherence using self-reporting in diaries and weekly follow-up phone calls. In IMT interventions, the number of home-based sessions was measured directly using the IMT device (12).

##### Frequency

The frequency and duration of programs varied from five sessions over a 1 week period (10) to three times per week for 8 weeks (26) with the exception of one study which occurred concurrently with neoadjuvant treatment and lasted up to 17 weeks (14). Interval training was utilised in six studies, with five prescribing high intensity interval cycling training (11, 28, 31, 32, 34) and one study including walking intervals (12).

##### Qualifications of Personnel

The providers of the intervention included a range of healthcare disciplines: physiotherapists (10, 12, 27, 33, 34, 36, 38, 39), kinesiologists (8, 13, 26, 29), physician (28), exercise science staff (31), study coordinators (14), trained fitness instructors (11, 15) or a combination (40). In one study the exercise intervention was prescribed by a physician and then demonstrated and monitored by a kinesiologist (32). Qualifications of personnel supervising the intervention were not reported in three studies (32, 35, 37). Thirteen (59%) of the studies were delivered individually (one-on-one) (12–15, 26–28, 30, 33, 34, 37–39, 41), while the remaining studies did not state how they were delivered.

##### Setting

Programs were most commonly delivered in a home-based setting (12, 13, 26, 30, 37, 39), hospital outpatient clinics (10, 27, 36), or a combination of hospital outpatient clinic and home-based settings (8, 33). Other sites included: exercise laboratories (15, 28, 29, 31, 32), rehabilitation centres (38), hospital research unit (11), combination of home-based and community (14), community program (34) or was not specifically reported (35, 40). Individualised exercise prescriptions were common in supervised exercise sessions however it was unclear in a number of cases whether the sessions were conducted individually (1:1) or as part of a group (8, 10, 11, 29, 31, 32, 35, 36, 40).

##### Adverse Events

Only one study (12) reported adverse events related to prehabilitation. The two events that were reported self-resolved and did not require any additional healthcare use, including musculoskeletal pain in pre-existing injuries and one episode of dizziness.

#### Nutritional Intervention

Nutritional interventions were included as part of multimodal prehabilitation in eight studies (8, 10, 13, 26, 29, 30, 39, 40) however reporting of the interventions varied widely in the included studies. Assessment for nutritional intervention was conducted by a registered dietitian individually in person in six studies (8, 13, 26, 29, 30, 40) and was unreported in the other studies (10, 39). Assessment focused on achieving daily dietary intake with a focus on target protein of between 1.2 and 1.5 g/kg of ideal body weight in six studies (8, 13, 26, 29, 30, 40) and involved whey protein supplement only in participants unable to achieve this with diet alone in three studies (8, 13, 29) and administered to all participants in two studies (26, 30) to ensure this target was being met. Whey protein supplements when included where recommended in the 1 h after exercise training to maximise protein synthesis (8, 13, 26, 29) or after breakfast on non-exercise training days (30).

Details of nutritional intervention in two other studies were non-specific and would not allow for replication with detail such as “liquid oral nutrition supplements and vitamin supplements” or “nutritional screening and counselling: supportive oral supplements when recommended” (10, 39, 40). Follow up of nutritional interventions was conducted by a nutritionist in four studies (8, 13, 29, 30). Only one study detailed weekly follow up phone calls by the nutritionist (30).

#### Psychological Intervention

Interventions aimed at reducing pre-operative anxiety (8, 13, 26, 29) as well as a motivational interview aimed at improving compliance with program elements (34) were incorporated as part of multimodal prehabilitation programs within included studies. Interventions at reducing pre-operative anxiety were delivered by a trained psychologist (26), a psychology trained nurse (8), a psychology-trained member of the research team (29) or not reported (13). The motivational interview was conducted by a specialised physiotherapist (34). All interventions were delivered as a one-off supervised session. Interventions aimed at reducing pre-operative anxiety included relaxation and imagery techniques. Participants were provided with an audio disc of exercises for home-based practise in three studies (8, 26, 29) and were encouraged to practise the techniques from daily (13) to three times per week (26, 29). However, adherence to these home based practise sessions was only incorporated into overall prehabilitation compliance by self-report in weekly phone calls in one study (26).

#### Education

Other educational elements included in multimodal prehabilitation programs included preoperative smoking and alcohol cessation information in three studies (8, 38, 39), however the reporting of who delivered this information, when, where and how was poor. Only one of the three studies reported that the information was delivered in person and individually by a psychology training nurse as part of the psychological intervention appointment (8).

#### Control Groups

Prehabilitation was compared to control groups which included standard care that included no prehabilitation intervention. Standard care varied across the included studies. Eleven studies included control groups with no intervention (11–14, 30, 32–35, 38, 39), three studies asked participants to maintain their current exercise and lifestyle habits (15, 28, 31) whereas three studies delivered the same multimodal intervention in the post-operative period instead of the preoperative period (8, 26, 29). Usual or standard care differed significantly amongst the remaining studies including physical activity recommendations (12), physical activity recommendation delivered in conjunction with nutrition counselling and advice for smoking cessation (10, 11, 36, 39) or breathing exercise information (27, 36) or was not standardised and according to local policies as in the case of a multicentre trial (33). When physical activity was recommended as part of usual care there were no limits placed on participants and participants were advised to follow clinical advice (32) and/or allowed to participate in any hospital or municipality-based exercise program (11).

### Functional Outcomes and Cardiopulmonary Fitness

**Figure 2:**
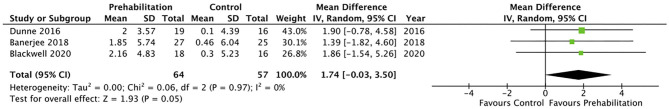
Meta-analysis of change in oxygen consumption (VO_2_) at peak (ml per kg per min) after prehabilitation.

**Figure 3:**
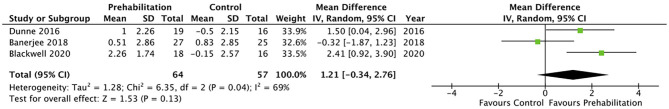
Meta-analysis of change in anaerobic threshold (AT) (ml per kg per min) after prehabilitation.

Five studies (28, 31, 32, 34, 36) measured cardiorespiratory fitness using oxygen consumption at peak exercise (VO_2_ peak). However, only three of the studies reported VO_2_ peak in comparable indices that allowed inclusion within the meta-analysis (Figure 2) (28, 31, 32). The meta-analysis of change in VO_2_ peak from baseline to after prehabilitation in these three studies (*n* = 121 participants) demonstrated a low quality evidence of improvement in cardiopulmonary fitness but did not achieve statistical significance (MD 1.74, 95% CI −0.03–3.50 mL/kg/min; *p* = 0.05; 95% PI −9.67 to 13.15; Figure 2). Of the studies that could not be included in the meta-analysis, one reported significantly increased (135%; *p* < 0.0001) endurance time with cycling at a constant work-rate at 80% of peak oxygen uptake (34) while the remaining study (36), which estimated maximal aerobic capacity using epidemiological data, reported no change after the exercise prehabilitation program (29.4 ± 9.5 to 27.6 ± 6.5 mL/kg/min; *p* = 0.47). Three studies (28, 31, 32) reported oxygen consumption at anaerobic threshold (AT) and meta-analysis demonstrated low quality evidence with no significant change after prehabilitation (MD 1.21, 95% CI −0.34–2.76 mL/kg/min; *p* = 0.13; 95% PI −16.33–18.75; Figure 3).

**Figure 4:**
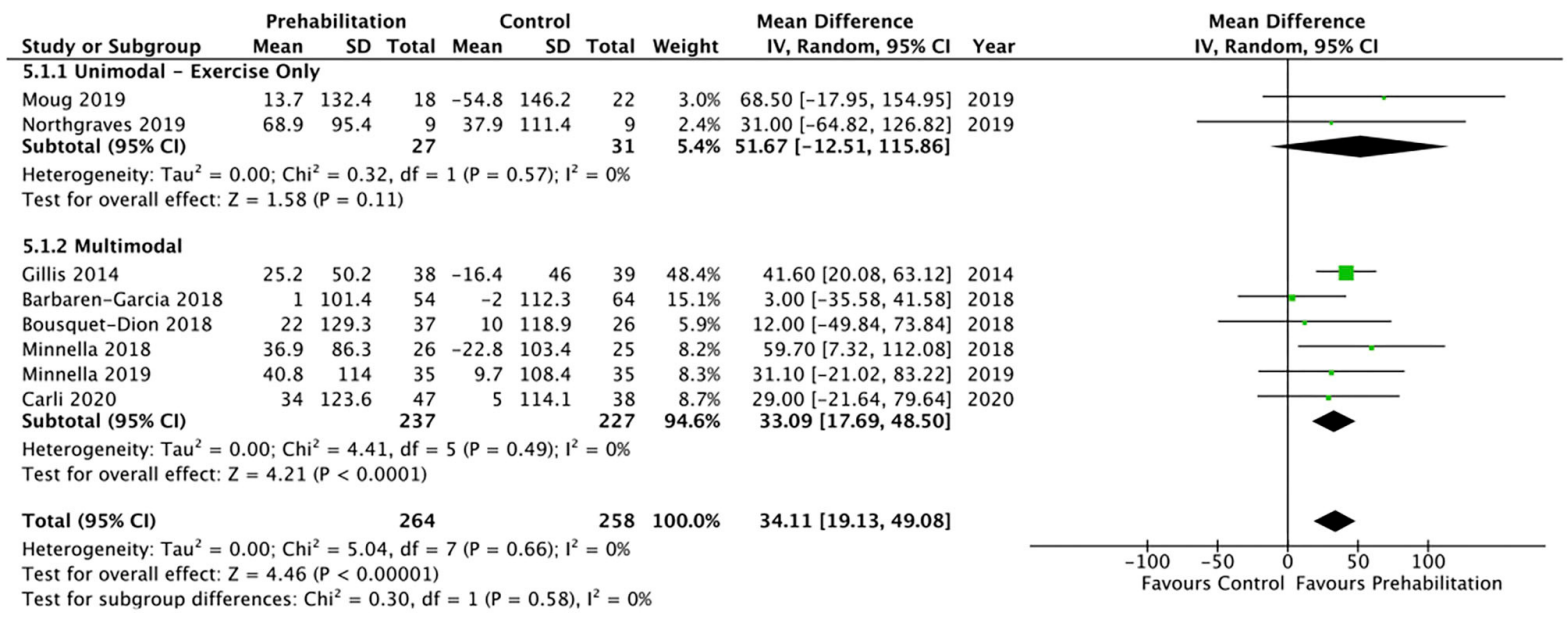
Meta-analysis of pre-surgical change in 6MWD (m) after prehabilitation.

Ten studies (8, 12–15, 26, 29, 30, 34, 35) reported data on functional exercise capacity using the 6MWT. Eight studies (8, 13–15, 26, 29, 30, 34) were included in the meta-analysis, showing moderate quality evidence of a favourable change in 6MWT following prehabilitation with a mean difference of 34.11 metres (95% CI 19.13–49.08; *p* < 0.1; 95% PI 15.42–52.80). Subgroup analysis of multimodal interventions (*n* = 6) demonstrated a favourable change in 6MWT of 33.09 metres (95% CI 17.69–48.50; *p* < 0.01; 95% PI 11.26–54.92) whereas unimodal interventions (*n* = 2) did not achieve significance of 51.67 metres (95% CI −12.51 to 115.86; *p* = 0.11; Figure 4). The timed up and go (TUG) was assessed in two studies with one small study finding a pattern of improvement after prehabilitation [mean difference of −0.44 s compared to the control group 0.36 s (15)] and the other finding no significant difference after prehabilitation (7.8 s, SD ± 3.3, *p* = 0.29) (36). The stair climbing test (SCT) and five times sit to stand (FTSTS) were assessed in the same small study as the TUG, with the SCT showing a favourable improvement (mean difference of −0.32 s compared to the control group 0.12 sec) whereas no pattern of improvement was reported in the FTSTS (15). Another study showed a 19% improvement in 10 metre walk test (10MWT) in the prehabilitation group, however this was not assessed against a control group for comparison (10).

### Postoperative Outcomes

**Figure 5:**
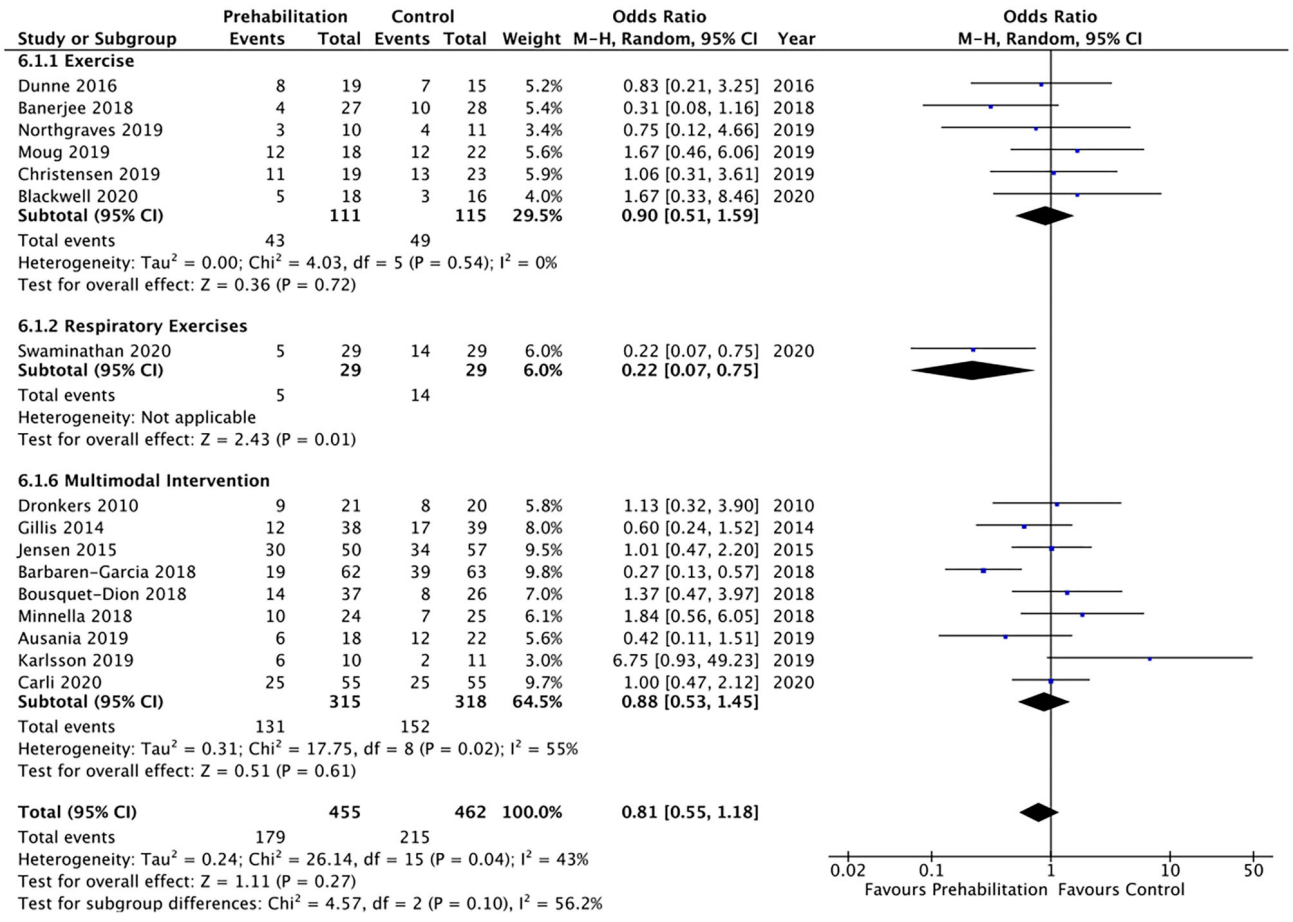
Meta-analysis of postoperative complications based on intervention group.

**Figure 6:**
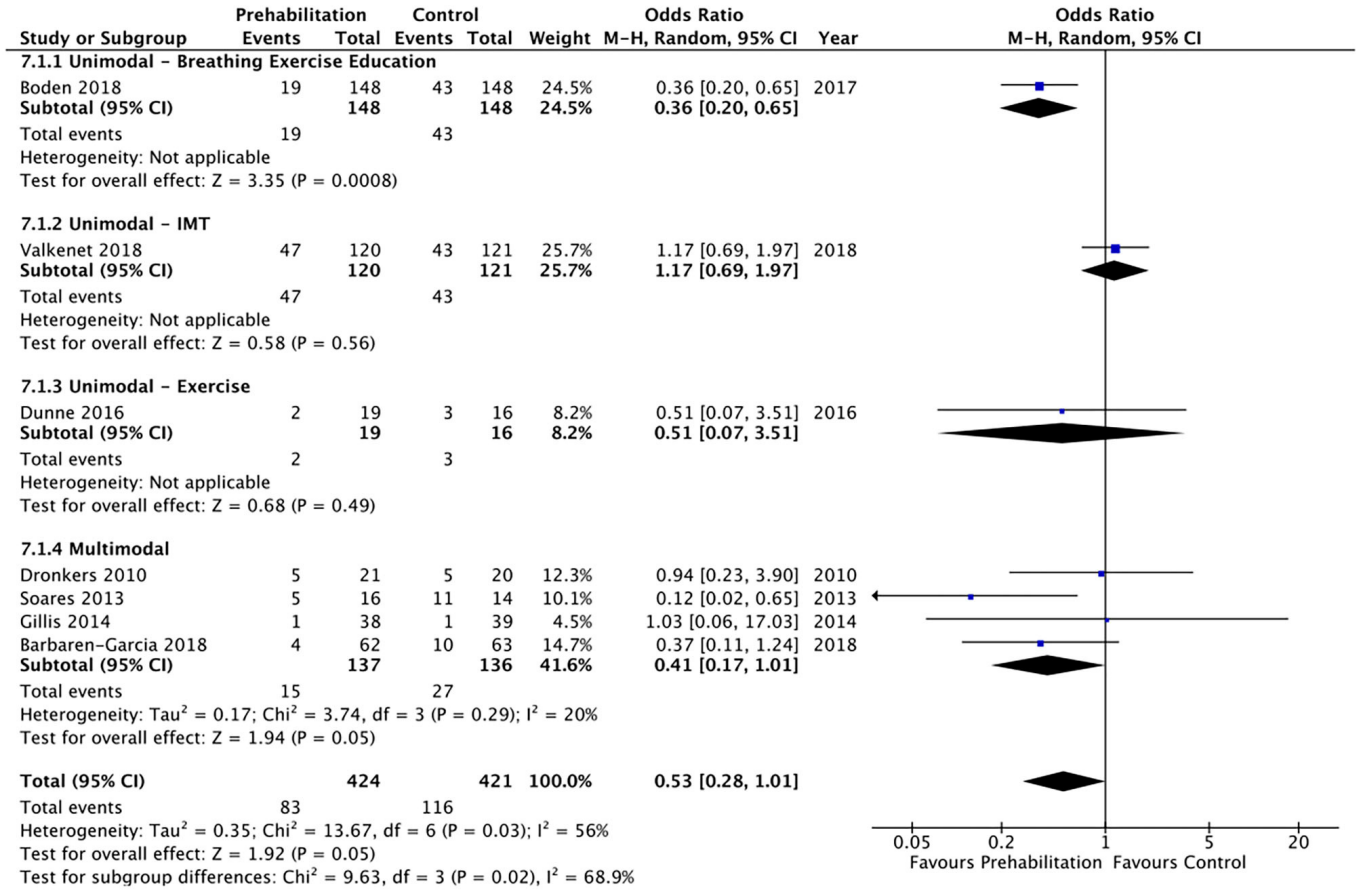
Meta-analysis of postoperative pulmonary complications based on intervention group.

Overall postoperative complications were measured in 16 studies and meta-analysis did not achieve significance (OR 0.81, 95% CI 0.55–1.18; *p* = 0.27; 95% PI 0.26–2.50; Figure 5). A trend was noted towards a reduction in postoperative pulmonary complications by prehabilitation, with the meta-analysis of the seven studies (26, 27, 32–36) that explored this endpoint, but this did not achieve statistical significance (OR 0.53, 95% CI 0.28–1.01; *p* = 0.05; 95% PI 0.09–3.02; Figure 6).

**Figure 7:**
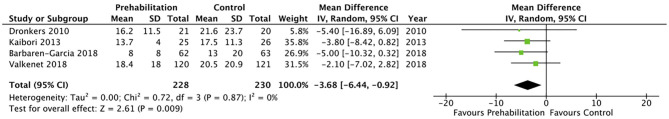
Meta-analysis of hospital length of stay (days).

**Figure 8:**
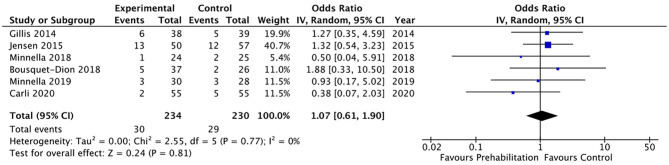
Meta-analysis of hospital re-admission after surgery.

**Figure 9:**
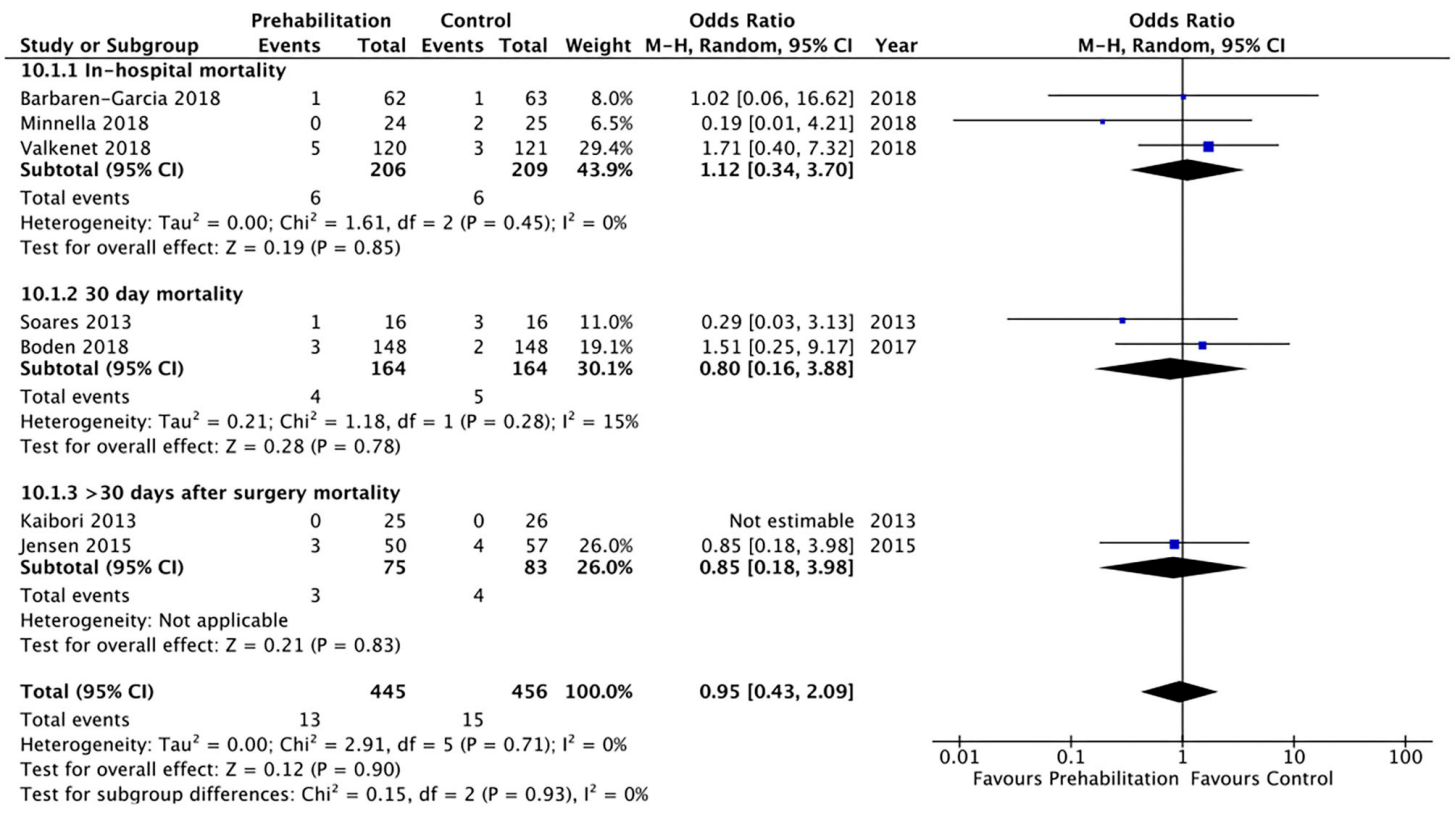
Meta-analysis of postoperative mortality.

Twenty of the included studies described hospital length of stay, however only four of the included studies (33, 34, 36, 40) reported data that was able to be included in a meta-analysis. The meta-analysis demonstrated moderate quality evidence favouring prehabilitation with a mean reduction of at least 3 days of hospital stay (MD 3.68, 95% CI 0.92–6.44; *p* = 0.009 and 95% PI −9.74 to 2.38; Figure 7). The meta-analysis of six studies (8, 13, 26, 29, 30, 39) showed moderate quality evidence with no significant difference in 30-day hospital re-admissions between prehabilitation and control groups (OR 1.07, 95% CI 0.61–1.90; *p* = 0.81 and 95% PI 0.47–2.41; Figure 8). Similarly, for the seven studies (27, 30, 33–35, 39, 40) that evaluated data on mortality the meta-analysis showed low quality evidence for no significant difference in mortality outcomes between patients that received prehabilitation or standard care (OR 0.95, 95% CI 0.43–2.09, *p* = 0.90, 95% PI 0.34–2.67; Figure 9).

### Risk of Bias and Quality of Evidence

**Figure 10:**
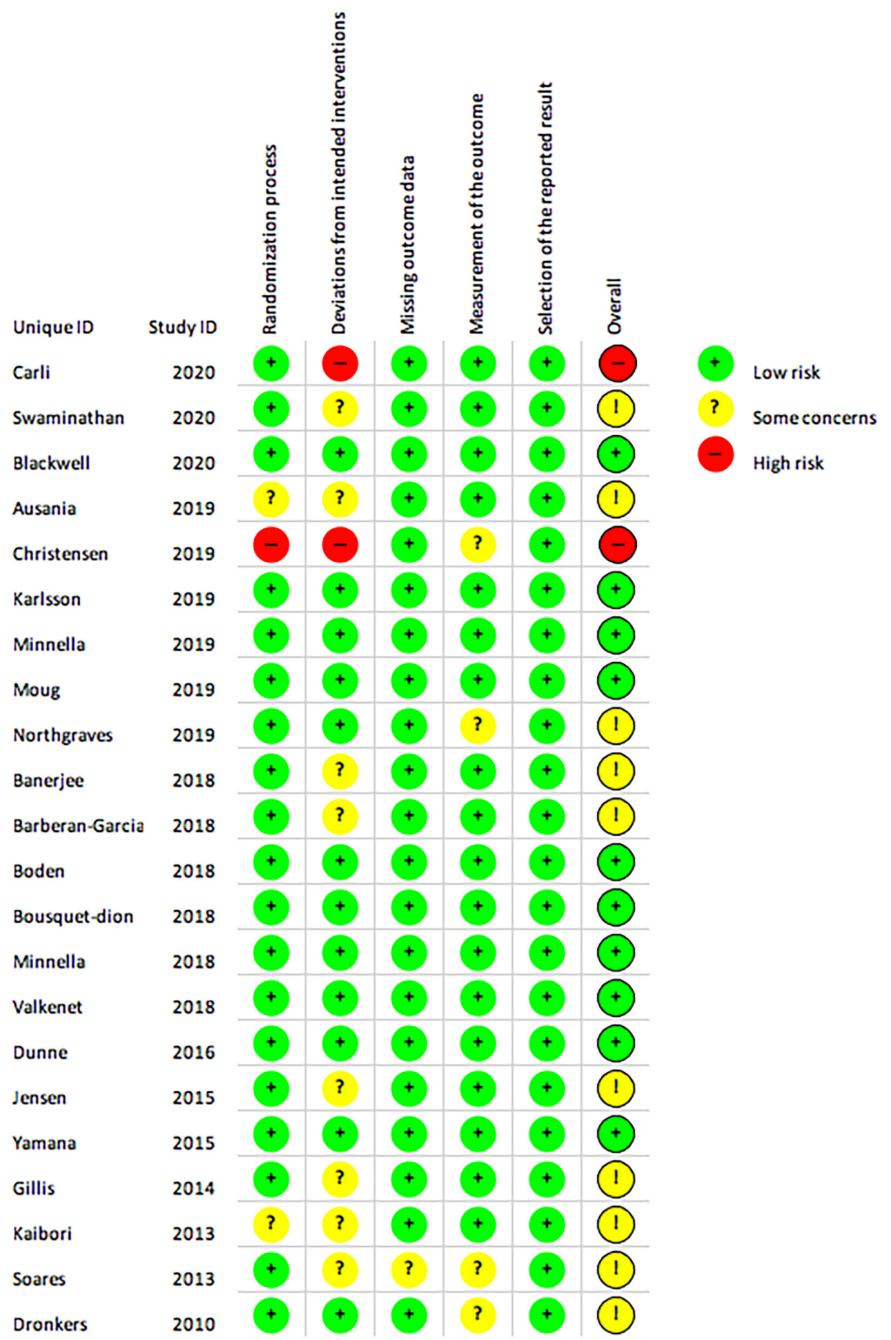
The Cochrane risk of bias assessment of randomised controlled trials. Green (+) = low risk; Yellow (?) = unclear risk; Red (-) = high risk.

Ten studies were assessed as having low risk of bias (Figure 10) (12–14, 27–30, 32, 33, 38). Two studies were assessed as having a high risk of bias (8, 11) due to pseudo-randomisation based on geographical locations (11) and due to the uneven adherence to programs in the intervention and control groups (8). The majority of studies were assessed as having some concern regarding the risk of bias, as although several of the studies were registered in clinical trial registries prior data analysis plans were not publicly available (13–15, 26, 28, 29, 31, 32, 34–40) however it was the authors judgement that this did not affect overall selection of reported result as this requirement has only been required for select journals in recent years. Quality of evidence evaluated using the GRADE approach are reported for meta-analyses of each outcome (Supplementary Tables 9, 10).

## Discussion

Alongside the growing literature base (6, 7, 42) and clinical recommendations (3) prehabilitation is being increasingly adopted into clinical practise to improve postoperative outcomes (4), especially for patients with cancer (3). This systematic review includes nine new RCTs (since the last review on this topic) (8, 10–15, 28, 37) and provides clinicians and policy makers with current research to inform future research directions and implementation strategies.

In this systematic review with meta-analysis we report that prehabilitation improves preoperative functional capacity and substantially reduces hospital length of hospital. Prehabilitation did not significantly change postoperative complications, 30-day hospital readmissions or postoperative mortality. However, these findings should be interpreted with caution, due to the substantial heterogeneity within and across the studies, small sample size of the included studies and incomplete reporting of exercise interventions. The willingness to participant must also be considered when interpreting findings as recruitment rates varied greatly within included studies and ranged from all patients approached consenting to participate (8) to as high as 65% (12) and 82% (15) of patients approached for inclusion declining to participate in prehabilitation. This acceptance may reflect individual aspects of the program suggested to influence patients waiting for cancer surgery such as delivery location, use of technology and recommendation by health professionals (43).

Improvements in surgical care, including the implementation of enhanced recovery after surgery (ERAS) pathway has added complexity to interpreting the efficacy of prehabilitation interventions in the post-operative period, particularly within the inclusion of early post-operative mobilisation and pain management which are likely to influence the development of PPCs and LOS. Our meta-analysis showed a reduction in LOS, but it should be noted none of studies included a formalised ERAS pathway (33, 34, 36, 40). A recently published multicentre trial of multimodal prehabilitation program found no reduction in postoperative outcomes including LOS in frail patients awaiting colorectal resection across two centres with already established ERAS pathways (8). However, research into prehabilitation in the frail cancer population is limited (8). Therefore, it remains unclear as to whether prehabilitation offers additional benefits when established ERAS pathways are already in place or whether prehabilitation needs to be more tailored (for example provided for a longer period) within the frail population to confer these added benefits. More research is needed to investigate the efficacy of prehabilitation on post-operative outcomes in centres with already established ERAS pathways.

Our meta-analysis demonstrates that multimodal prehabilitation including exercise (combined aerobic and resistance training), nutritional intervention and anxiety reduction strategies, but not unimodal exercise, prehabilitation interventions, result in a clinically significant improvements in functional capacity as measured by 6 min walk distance (6MWD) (44). This differs from a recent systematic review conducted by Hughes et al. (45) who reported no significant change in pre-operative 6MWD, however this was only conducted on three studies and the availability of more recently published studies likely contributed to this difference (13–15, 29, 30, 34). The mean change in 6MWD was 33 metres with confidence intervals between 18 and 49 metres. This is a clinically relevant improvement when compared with MCID of lung cancer populations (46) of between 20 and 40 metres. There is currently no specific value reported for the abdominal surgical cancer population.

We report a trend towards improved cardiopulmonary fitness but the improvement did not reach significance. This may reflect on a limited number of studies included in the meta-analysis and underpowering of the included studies for these CPET-derived endpoints. There is a need to reflect on the heterogeneity of exercise interventions and compliance to achieve effective prehabilitation and underlying disease state that precludes some patients from responding to prehabilitation. The use of reporting templates for exercise interventions such as the CERT (20) would assist in more detailed information that could be pooled for meta-analysis as well as replication in research and implementation into clinical care (47). Huang et al. (48) reported that in those patients who were referred to a prehabilitation program there were a number of non-responders to prehabilitation. This warrants further investigation, exploring ways to improve the effectiveness of prehabilitation programs but also the importance of understanding the impact of the underlying disease state e.g., cancer associated inflammation and its associated therapies e.g., neoadjuvant chemoradiotherapy to identify responders.

There remains ongoing debate regarding the most suitable CPET variables for surgical risk prediction and for monitoring effective prehabilitation (49). A recent systematic review advocates that high-intensity (75–80% of max) constant work rate may provide increased sensitivity to changes in fitness as a result of prehabilitation (50). More importantly, is what type of exercise should be utilised within the more superior multimodal prehabilitation programs to be effective within the short timeframe that is available prior to surgery. A recent study found similar improvements in preoperative functional capacity using high-intensity interval training (HIIT) compared to moderate intensity continuous training (MICT), however at 2 months after surgery the HIIT group sustained greater physical fitness. The role of multimodal prehabilitation, that includes exercise, psychological and nutritional input is supported by this meta-analysis.

In contrast to recent reviews (45, 51) no difference was found in all-cause postoperative complications or postoperative pulmonary complications. Individual studies showed mixed results with pre-operative respiratory education (27) and multimodal interventions including exercise, IMT and respiratory exercises (35) eliciting significant reductions in postoperative pulmonary complications, whereas IMT alone was not significant (33). However, there is a lack of consistency regarding outcome measures used and timing of their application to measure the impact on postoperative complications. This prevented synthesis of findings from a greater number of studies included in the review. Abbott et al. (41) published a consensus on standardising these endpoints for pulmonary complications to enhance perioperative research, including a new definition of postoperative pulmonary complication which incorporates a measure of severity. Although many of the studies included in this meta-analysis were already in progress prior to its publication. It is promising to note that time and effort is being directed towards the standardisation of outcome measures in perioperative care research (52). However, the development of a core (minimum) set of outcome measures by multidisciplinary healthcare professionals, researchers and consumers with experience in prehabilitation will be essential to strengthen this literature base going forward.

Multimodal prehabilitation programs are increasingly implemented into standard care (4) and a multimodal approach is advised based upon results of this review (2, 3, 53). There are several large RCTs, aiming to recruit between 154 and 1,560 participants, currently in progress that will continue to strengthen this literature base (54–57). These studies include an international multicentre multimodal prehabilitation intervention including exercise, nutrition and psychological coping strategies within an ERAS protocol (Trial ID NTR5947) (55) as well as an in depth look at preoperative exercise setting by comparing hospital based supervised exercise, supported home based exercise vs. usual care in a 3-arm RCT (Trial ID ISRCTN82233115) (56), an investigation of the effectiveness of a community based prehabilitation intervention including a structured responsive exercise training program with or without psychological support (Trial ID NCT03509428) (57) as well as investigating the effectiveness of preoperative IMT (Trial ID ISRCTN10644366) (58). However, there seems to remain a blanket approach to prehabilitation despite the fact that certain groups may benefit more greatly (59) or have increasing needs (8). It may be that a stepped care model of prehabilitation (3), may be more cost effective where higher risk patients receive targeted and intensive individualised interventions and low risk patients receive more generalised universal interventions such as preoperative education, such as the approach used by Moore et al. in the implementation of a “Prehab4Cancer” program (4).

### Future Directions

Much of the prehabilitation literature focuses on the immediate postoperative course of patients with certain studies focusing on functional recovery up until 8 weeks postoperatively (13, 26, 30, 60). However, there is a lack of research into how this affects return to intended oncologic (adjuvant) therapies and ongoing exercise behaviour. These offer exciting avenues of research in the future. Future research should also investigate which aspects of prehabilitation may be more effective, type and intensity of exercise, delivery settings, impact on higher risk subgroups such as the frail or elderly, impacts of biological outcomes such as inflammatory markers, the additional use of newer technologies, the cost effectiveness of prehabilitation as well as the ability to ensure patients are fit enough to withstand treatment, discharge from hospital and return as soon as possible to intended oncologic therapies. Furthermore, standardisation of outcome measures is needed to allow researchers to inform meta-analyses more effectively. This minimises research waste and allows analysis of larger sample sizes (61, 62). Results from future studies will in turn provide guidance for clinicians and health services who provide prehabilitation and expediate implementation of prehabilitation into practise and policy.

This review benefits from robust methods in keeping with established guidelines (25), including a registered protocol. Searches were comprehensive and screening, data extraction and quality appraisal conducted in duplicate as well as exercise interventions reported according to clinical consensus guidelines (20). However, potential limitations associated with our systematic review methodology may be the restriction to studies published after Jan 1st, 2010 and the exclusion of unimodal non-exercise interventions. However, the rapidly growing area of prehabilitation warranted a focus on the most up to date literature within the context of current surgical practises. Studies were also restricted to English; however, no articles were excluded at title and abstract screening stage that appeared potentially relevant to this language restriction. The principal limitations of the findings of this study are the heterogeneity of the types of interventions and the outcome measures used to assess the effects of prehabilitation. There was also an inability to retrieve data, in response to author request, and data had to be calculated or inferred from study figures for inclusion in the meta-analysis.

In conclusion, this systematic review demonstrated that prehabilitation improved preoperative functional exercise capacity after multimodal prehabilitation programs with a reduction in hospital length of stay. These findings should however be interpreted with caution, given the heterogeneity of included studies. Never-the-less, these promising results warrant larger efficacy studies and cost-effectiveness studies.

## Data Availability Statement

The original contributions presented in the study are included in the article/Supplementary Material, further inquiries can be directed to the corresponding author/s.

## Author Contributions

Concept, idea and research design were conducted by JW, LD, BR, and CG. Writing by JW, LD, LE, CG, and BR. Data collection by JW, OM, and LE. Data analysis by JW. Data interpretation by JW, LD, HI, LE, and BR. All authors contributed to the critical review of the manuscript before submission.

## Conflict of Interest

The authors declare that the research was conducted in the absence of any commercial or financial relationships that could be construed as a potential conflict of interest.
