# Supplementary material for: Efficacy of Prehabilitation Including Exercise on Postoperative Outcomes Following Abdominal Cancer Surgery: A Systematic Review and Meta-Analysis
Source: Front Surg. 2021 Mar 19;8:628848. doi: 10.3389/fsurg.2021.628848 (PMC8017317; doi:10.3389/fsurg.2021.628848)
Supplement: Supplementary file 1 [file Data_Sheet_1.docx]

Supplementary Material

# Database Searches

## Database: Ovid MEDLINE(R) and Epub Ahead of Print, In-Process & Other Non-Indexed Citations and Daily <1946 to August 14, 2020>

Search Strategy:

--------------------------------------------------------------------------------

1 Preoperative Period/ or Preoperative Care/ or preoperati*.mp.
2 surgical*.mp.
3 Surgical Procedures, Operative/
4 operati*.mp.
5 1 or 2 or 3 or 4
6 Neoplasms/ or neoplas*.mp.
7 Abdominal Neoplasms/ or Carcinoma/ or abdominal neoplasm*.mp.
8 carcinoma*.mp.
9 tumour*.mp.
10 tumor*.mp.
11 cancer*.mp.
12 oncolog*.mp.
13 malignan*.mp.
14 metasta*.mp.
15 colorectal*.mp.
16 rectal*.mp.
17 6 or 7 or 8 or 9 or 10 or 11 or 12 or 13 or 14 or 15 or 16
18 Exercise Therapy/
19 exercise*.mp.
20 exercise program*.mp. or Physical Fitness/
21 Physical Therapy Modalities/
22 resistance training.mp. or Resistance Training/ or "Physical Education and Training"/
23 strength training.mp.
24 High-Intensity Interval Training/
25 interval training.mp.
26 high-intensity intermittent exercise.mp.
27 physical activit*.mp.
28 exercise therap*.mp.
29 physical therap*.mp.
30 physiotherap*.mp.
31 health-related fitness.mp.
32 prehab*.mp.
33 Nutrition Therapy/
34 nutrition therap*.mp.
35 nutrition intervent*.mp.
36 Stress, Psychological/
37 Resilience, Psychological/
38 Smoking Cessation/
39 Alcohol Drinking/ or alcohol intake.mp.
40 Dietary Supplements/

41 18 or 19 or 20 or 21 or 22 or 23 or 24 or 25 or 26 or 27 or 28 or 29 or 30 or 31 or 32 or 33 or 34 or 35 or 36 or 37 or 38 or 39 or 40
42 Postoperative Com
44 "Recovery of Function"/ or function* recovery.mp.
45 morbidity/ or mortality/ or fatal outcome/ or hospital mortality/ or mortality, premature/
46 "length of stay"/ or patient readmission/
47 length of stay.mp.
48 ICU admission.mp.
49 outcome measure*.mp.
50 patient outcome*.mp.
51 42 or 43 or 44 or 45 or 46 or 47 or 48 or 49 or 50
52 5 and 17 and 41 and 51
53 limit 52 to yr="2010 -Current"

***************************

## Database: Embase Classic+Embase <1947 to 2020 Week 33>

Search Strategy:

--------------------------------------------------------------------------------

1 preoperative care/
2 preoperative period/
3 preoperati*.mp.
4 neoplasm/
5 cancer*.mp.
6 oncolog*.mp.
7 malignan*.mp.
8 metasta*.mp.
9 colorectal*.mp.
10 rectal*.mp.
11 abdominal neoplasm.mp. or abdominal tumor/
12 exercise/ or aerobic exercise/ or breathing exercise/ or high intensity interval training/ or resistance training/
13 physical therapy modalities.mp. or physiotherapy/
14 exercise*.mp.
15 physical activity/
16 physical activit*.mp.
17 kinesiotherapy/
18 exercise therap*.mp.
19 physiotherap*.mp.
20 physical therap*.mp.
21 fitness/
22 resistance training.mp.
23 strength training.mp.
24 exercise program*.mp.
25 interval training.mp.
26 high-intensity intermittent exercise.mp.
27 prehab*.mp.
28 diet therapy/
29 nutrition therap*.mp.
30 mental stress/
31 psychological resilience/
32 smoking cessation/
33 drinking behavior/
34 alcohol consumption/
36 postoperative complications.mp. or postoperative complication/
37 complication/ or postoperative complication/
38 function* recovery.mp.
39 morbidity/
40 cancer mortality/ or mortality.mp. or all cause mortality/ or mortality/ or surgical mortality/
41 length of stay.mp. or "length of stay"/
42 ICU admission.mp.
43 outcome measure*.mp.
44 treatment outcome/
45 patient outcome*.mp.
46 36 or 37 or 38 or 39 or 40 or 41 or 42 or 43 or 44 or 45
47 abdominal surgery/ or surgery/
48 1 or 2 or 3 or 47
49 4 or 5 or 6 or 7 or 8 or 9 or 10 or 11
50 35 and 46 and 48 and 49
51 limit 50 to yr="2010 -Current"
52 diet supplementation/ or dietary supplement/
53 12 or 13 or 14 or 15 or 16 or 17 or 18 or 19 or 20 or 21 or 22 or 23 or 24 or 25 or 26 or 27 or 28 or 29 or 30 or 31 or 32 or 33 or 34 or 52
54 46 and 48 and 49 and 53
55 limit 54 to yr="2010 -Current"

***************************

## Database: CINAHL

Search Strategy:

--------------------------------------------------------------------------------

| **#** | **Query** |
| --- | --- |
| S56 | S9 AND S24 AND S42 AND S55 |
| S55 | S43 OR S44 OR S45 OR S46 OR S47 OR S48 OR S49 OR S50 OR S51 OR S52 OR S53 OR S54 |
| S54 | "outcome measure*" |
| S53 | "patient outcome*" |
| S52 | "ICU admission" |
| S51 | "length of stay" |
| S50 | (MH "Length of Stay") |
| S49 | (MH "Functional Status") |
| S48 | "functional recovery" OR recovery |
| S47 | (MH "Outcomes (Health Care)") |
| S46 | (MH "Morbidity") OR (MH "Mortality") OR (MH "Hospital Mortality") |
| S45 | complication* |
| S44 | "post-operative complication*" |
| S43 | (MH "Postoperative Complications") |
| S42 | S25 OR S26 OR S27 OR S28 OR S29 OR S30 OR S31 OR S32 OR S33 OR S34 OR S35 OR S36 OR S37 OR S38 OR S39 OR S40 OR S41 |
| S41 | "alcohol intake" |
| S40 | (MH "Alcohol Drinking") |
| S39 | (MH "Smoking Cessation") |
| S38 | (MH "Stress, Psychological") |
| S37 | "nutrition therap*" OR "nutrition intervention*" OR "nutrition education" |
| S36 | (MH "Diet Therapy") |
| S35 | "interval training" OR "high intensity intermittent exercise" OR "prehab*" |
| S34 | "resistance training" OR "strength training" OR "exercise program*" |
| S33 | "health-related fitness" |
| S32 | "exercise therap*" OR "physical therap*" OR physiotherap* |
| S31 | (MH "Therapeutic Exercise") |
| S30 | "physical activit*" |
| S29 | exercis* |
| S28 | (MH "Preoperative Education") OR (MH "Patient Education") |
| S27 | (MH "Resistance Training") |
| S26 | (MH "Physical Therapy") OR (MH "Rehabilitation, Cancer") |
| S25 | (MH "Exercise") OR (MH "Aerobic Exercises") OR (MH "High-Intensity Interval Training") OR (MH "Muscle Strengthening") OR (MH "Resistance Training") OR (MH "Cardiorespiratory Fitness") OR (MH "Physical Fitness") |
| S24 | S10 OR S11 OR S12 OR S13 OR S14 OR S15 OR S16 OR S17 OR S18 OR S19 OR S20 OR S21 OR S22 OR S23 |
| S23 | "abdominal neoplasm*" |
| S22 | rectal* |
| S21 | colorectal* |
| S20 | carcinogen* |
| S19 | metasta* |
| S18 | malignan* |
| S17 | oncolog* |
| S16 | cancer* |
| S15 | tumor* |
| S14 | tumour* |
| S13 | neoplasm* |
| S12 | carcinoma* |
| S11 | (MH "Carcinoma") |
| S10 | (MH "Abdominal Neoplasms") OR (MH "Neoplasms") |
| S9 | S1 OR S2 OR S3 OR S4 OR S5 OR S6 OR S7 OR S8 |
| S8 | invasive* |
| S7 | preoperati* |
| S6 | operation* |
| S5 | surgical* |
| S4 | surger* |
| S3 | (MH "Surgery, Operative") |
| S2 | (MH "Laparotomy") OR (MH "Pelvic Exenteration") OR (MH "Preoperative Period") OR (MH "Surgery, Digestive System") OR (MH "Surgery, Elective") |
| S1 | (MH "Prehabilitation") OR (MH "Preoperative Care") OR (MH "Preoperative Education") |

## Database: PEDro

Search Strategy

Prehab*, year 2010 to current

Abstracts 36

Preoperative, year 2010 to current in oncology subdiscipline

Abstracts 81

## Database: EBM Reviews - Cochrane Central Register of Controlled Trials <July 2020>

**Search Strategy:**

--------------------------------------------------------------------------------

1 Preoperative Period/ or Preoperative Care/ or preoperati*.mp.
2 surgical*.mp.
3 Surgical Procedures, Operative/
4 operati*.mp.
5 1 or 2 or 3 or 4
6 Neoplasms/ or neoplas*.mp.
7 Abdominal Neoplasms/ or Carcinoma/ or abdominal neoplasm*.mp.
8 carcinoma*.mp.
9 tumour*.mp.
10 tumor*.mp.
11 cancer*.mp.
12 oncolog*.mp.
13 malignan*.mp.
14 metasta*.mp.
15 colorectal*.mp.
16 rectal*.mp.
17 6 or 7 or 8 or 9 or 10 or 11 or 12 or 13 or 14 or 15 or 16
18 Exercise Therapy/
19 exercise*.mp.
20 exercise program*.mp. or Physical Fitness/
21 Physical Therapy Modalities/
22 resistance training.mp. or Resistance Training/ or "Physical Education and Training"/
23 strength training.mp.
24 High-Intensity Interval Training/
25 interval training.mp.
26 high-intensity intermittent exercise.mp.
27 physical activit*.mp.
28 exercise therap*.mp.
29 physical therap*.mp.
30 physiotherap*.mp.
31 health-related fitness.mp.
32 prehab*.mp.
33 Nutrition Therapy/
34 nutrition therap*.mp.
35 nutrition intervent*.mp.
36 Stress, Psychological/
37 Resilience, Psychological/
38 Smoking Cessation/
39 Alcohol Drinking/ or alcohol intake.mp.
41 Postoperative Complications/ or post operative complications.mp.
42 complications.mp.
43 "Recovery of Function"/ or function* recovery.mp.
44 morbidity/ or mortality/ or fatal outcome/ or hospital mortality/ or mortality, premature/
45 "length of stay"/ or patient readmission/
46 length of stay.mp.
47 ICU admission.mp.
48 outcome measure*.mp.
49 patient outcome*.mp.
50 41 or 42 or 43 or 44 or 45 or 46 or 47 or 48 or 49
51 5 and 17 and 40 and 50
52 limit 51 to yr="2010 -Current"
53 general surgery/ or surgical oncology/
54 2 or 3 or 4 or 53
63 Dietary Supplements/
64 18 or 19 or 20 or 21 or 22 or 23 or 24 or 25 or 26 or 27 or 28 or 29 or 30 or 31 or 32 or 33 or 34 or 35 or 36 or 37 or 38 or 39 or 63
65 5 and 17 and 50 and 64
66 limit 65 to yr="2010 -Current"

***************************

# Consensus on Exercise Reporting Template [1]

Exercise interventions are complex, varying greatly in their mode, frequency, duration, intensity, equipment and individualisation. However, descriptions within the literature can be vague and simply state exercise modality ‘strengthening’. This lack of detail can potentially lead to compromised evaluation, replication and implementation into clinical practice. Research prior to the development of the CERT for reporting exercise interventions Hoffman and colleagues [2] found that only 39% of non-pharmacological interventions were described in a way that allowed replication. When studying interventions for low back pain, Gianola and colleagues [3] found that when describing the intervention ‘less than one fifth would be replicable clinically’.

The CERT was developed by an international panel of exercise professionals to establish a consensus on reporting exercise interventions that would not only improve transparency of the research but also aloe exercise interventions to be replicated and implemented into clinical care. The CERT is divided into 7 domains; materials, provider, delivery, location, dosage, tailoring and compliance.

The CERT is now recommended by the EQUATOR Network (Enhancing the Quality and Transparency of Health Research; www.equator-network.org) and the International Journal of Sports Physical Therapy [4] to improve the reporting of therapeutic exercise interventions in rehabilitation research.

# Prediction Interval [5]

It has been suggested that the previous use of the inconsistency (I^2^) statistic to assess heterogeneity within systematic reviews may be improved by changing the index used to assess this variation across studies. Whereas the I^2^ estimates the observed effect size in the study’s sample, which may under-estimate or over-estimate the true effect size that may exist within the entire population due to sampling error, the prediction interval estimates the true effect size for each included study. I^2^ is also a proportion, not an absolute value. The prediction interval is a clear and reported on the same scale as the effect size. Utilising the prediction interval means a few conditions must be met. There must be a sufficient number of studies. These studies must be random (or at least representative) sample of the intended universe. It is also assumed that the effects are normally distributed on the relevant scale. There is no consensus on the number of studies needed as a minimum to utilise this index however a higher number of studies will be more appropriate in discussing the utility of an intervention.

# 4 Table 1 – Outcomes According to Prehabilitation Timepoint

|  | **Functional Measures** | | | |  |  |  | **Post-Operative Outcomes** | | |  | |
| --- | --- | --- | --- | --- | --- | --- | --- | --- | --- | --- | --- | --- |
| **Study** | **Baseline** | **Pre-surgery** | **Post-op** | | **Prehab program designed (weeks)** | **Prehab delivered (days)** | **Time from baseline to surgery(days)** | **Complications, n(%)** | **Hospital Length of Stay, days** | **Readmission, n(%)** | **Morality, n(%)** |  |
| **6MWD** |  |  |  | |  |  |  |  |  |  |  |  |
| **Carli 2020** | 6MWD I: 325.2±114.3 C: 304.0±107.3 | 6MWD I: 345.1±117.8 C: 315.8±107.5 Mean diff: 11.2 95%CI (-13.7 to 36.1) p=0.37 | 6MWD (4) I: 336.4±121.8 C: 286.1± 105.1 Mean diff: 18.5 (-20.2 to 57.3) p=0.34 | | 4 | NR | I:40[28-51] C:35[22-55] | I: 25(45.5%) C: 25(45.5%) OR: 0.9(0.4 to 2.2), p=0.90  CCI I: 12.7±21.5  C: 15.7±25.3 Mean diff -3.2(-11.8 to 5.3), p=0.45 | I: 4[3-8] C: 4[3-8] HR: 19.2(-2.9 to 2.2) p=0.80 | I: 2(3.6%) C:5(9.1%) OR: 0.3(0.02 to 1.9), p=0.18 |  |  |
| **Karlsson 2019** | 6MWD, median(95%CI) I: 418.5(300-531)  C:432(357-476)  30STS I:11(6-14) C:11(8-15) | 6MWD I:398(279-553), change 15 (-29,46), p=NS C:426(380-482), change  -4 (-16,20), p=NS  30STS I:13.6(10-16), change +3.5 (0,4), p=NS C:12(9-16), change +1 (-0.3,3.3), p=NS | 6MWD (hospital discharge) I:330(241-448), p=0.03 C: 278.5(115-402), p=0.003  30STS I:11(1-13), p=NS C:11.5(4-14), p=0.02 | | 2-3 | NR | I: 17(14-24) C: 14 | Any postop complications, n (%) I: 6 (60.0%) C:2 (18.2%), p=0.06 | I: 5[4-6] C: 6[4-7], p=0.57 |  |  |  |
|  | **Functional Measures** | | | |  |  |  | **Post-Operative Outcomes** | | | |  |
| **Study** | **Baseline** | **Pre-surgery** | **Post-op** | | **Prehab program designed (weeks)** | **Prehab delivered (days)** | **Time from baseline to surgery(days)** | **Complications, n(%)** | **Hospital Length of Stay, days** | **Readmission, n(%)** | **Morality, n(%)** |  |
| **Minnella 2019** | 6MWD I:399.4±141.3 C:421.8±148.0 | Change compared to baseline 6MWD I:40.8±114.0 C:9.7±108.4 p=0.250 | 6MWD (4): Change compared to baseline I: -15.4± 142.5 C: -97.9±123.8 p=0.014  6MWD (8): Change compared to baseline I: -5.6±173.5 C: -35.5±131.8 p=0.422 | | Baseline until surgery, | 27[19.8-44.5] | 27[19.8-44.5] | CD: p=0.528 I: 14(46.7%), C: 12(42.9%)   1. I: 4(13.3%), C: 4(12.3%) 2. I:9(30.0%), C: 8(28.6%)  - IIIa) I: 0(0%), C: 1(3.6%) - IIIb) I: 1(3.3%), C: 3(10.7%)   Iva) I: 3(10%), C: 3(10.7%) | I: 9.0[7.0-15.0] C:10.0[7.5-14.5] p=0.360 | 30 day I: 3(10%) C:3(10.7%) p=0.929 |  |  |
| **Moug 2019** | 6MWD I:448.8±64.9 C:444.9±59.2  30STS I:11.5±2.5 C:11.5±3.0 | 6MWD I:462.5±144.3 C:390.1±159.4  Group difference, mean(95%CI) 68.5(-27.2, 164.2)  30STS I:11.1±4.2 C:11.7±6.1 | N/A | | Baseline until surgery | 14[13-17] (0-17) * |  | Total:24(60%) I:12(67%) C:12(55%) | Median(range) Total:10.5(0.0-38.2) I:11.0(6.0-37.0) C:10(0.0-38.2) |  |  |  |
|  | **Functional Measures** | | | |  |  |  | **Post-Operative Outcomes** | | | |  |
| **Study** | **Baseline** | **Pre-surgery** | **Post-op** | | **Prehab program designed (weeks)** | **Prehab delivered (days)** | **Time from baseline to surgery(days)** | **Complications, n(%)** | **Hospital Length of Stay, days** | **Readmission, n(%)** | **Morality, n(%)** |  |
| **Northgraves 2019** | 6MWD  I: 404.8±80 C:422.8±97  TUG I:6.79±1.46 C:6.83±1.49  SCT I:2.79±0.61 C:2.91±0.50  FTSTS I:11.37±2.16 C:11.95±1.65 | 6MWD I: 473.7±93 C:460.7±106 Mean difference I: 68.9±37.6 C:7.9±38.6  TUG I:6.35±1.63 C:7.18±1.55 Mean difference  I: -0.44±0.35 C:0.36±0.66  SCT I:2.47±0.46 C:3.02±0.72 Mean difference  I: -0.32±0.18 C:0.12±0.33  FTSTS I:10.71±2.57 C:11.42±3.01 Mean difference  I: -0.66±0.92 C: -0.54±1.62 | N/A | | Baseline until surgery, | 22±7.5 (13-35) |  | I:3(30%) C:4(36%) | I:10[7](5-12) C:8[5](6-27) median difference 1 (95% CI -3,6) |  |  |  |
|  | **Functional Measures** | | | |  |  |  | **Post-Operative Outcomes** | | | |  |
| **Study** | **Baseline** | **Pre-surgery** | | **Post-op** | **Prehab program designed (weeks)** | **Prehab delivered (days)** | **Time from baseline to surgery(days)** | **Complications, n(%)** | **Hospital Length of Stay, days** | **Readmission, n(%)** | **Morality, n(%)** |  |
| **Barberan-Garcia 2018** | 6MWD I: 472±94 C: 471±95  CPET End. Time, s I: 325±151 C: 323±168 | 6MWD I: 473±91, p=0.953 C: 469±109, p=0.804  CPET End. Time, s I: 765±395, p=<0.001 C: 362±215, p=0.118 | | N/A | 6±2 | NR |  | I: 19 (31%) C: 39 (62%) RR 0.5  95%CI 0.3-0.8 p=0.001 | I: 8±8 C: 13±20, p=0.078  ICU I: 1±2 C: 4±13, p=0.078 |  | Morality I:1(2%) C:1(2%) p=1.000 |  |
| **Bousquet-Dion 2018** | 6MWD I: 448±118 C: 461±109 | 6MWD I: 470±118 C: 471±108 | | 6MWD (4) I: 441±120 C: 444± 116  6MWD (8) I: 468±118 C: 472±108 | 4 | NR | I: 32[25-48] C: 20.5[15-32] | I: 14(38%) C: 8(31%) p=0.562 | I:3[3-5] C:3[2-4] p=0.111 | 30 day I: 5 C: 2 p=0.415 |  |  |
| **Minnella 2018** | 6MWD I: 452.1±83.4 C: 449.2±83.9, p=0.43 | 6MWD I: 489.0±73.5 C: 426.4±102.7, p=0.02 | | 6MWD (4-8) I: 481.5±81.5 C: 379.8±106.0, p<.001 | Baseline until surgery | 36[17-73] | C: 51[12-71] | I:10(42%) (n=24) C:7(28%) (n=25)  I I: 2 (8) C: 0 II I: 6 (25) C: 8 (32) IIIa I: 3 (13) C: 7 (28) Iva I: 2 (8) C: 1 (4) Ivb I: 1 (4) C: 0 V I: 0 C: 2 (8), p=.23  CCI: I:14.8[0.0-28.8] C:20.9[20.9-36.2] | I:8.0[5.75-11.75] C:7.0[5.5-12.5] p=0.44 | I:1(4%) C:2(8%) | In-hospital mortality: I:0 C:2(8) |  |
|  | **Functional Measures** | | | |  |  |  | **Post-Operative Outcomes** | | | |  |
| **Study** | **Baseline** | **Pre-surgery** | **Post-op** | | **Prehab program designed (weeks)** | **Prehab delivered (days)** | **Time from baseline to surgery(days)** | **Complications, n(%)** | **Hospital Length of Stay, days** | **Readmission, n(%)** | **Morality, n(%)** |  |
| **Gillis 2014** | 6MWD I: 421±120 C:425±84 | 6MWD I: 450 C:410 p<0.001  Mean change in 6MWT±SD preop, m I: 25.2±50.2 C: -16.4±46, p=<.001 | 6MWD(4) I: 410 C:380  6MWD(8) I:450 C:400  Mean change in 6MWT±SD 8weeks postop, m I: 23.4±54.8 C: -21.8±80.7, p=.020 | | Baseline until surgery | 24.5[20-35] | I: 24.5[20-35] C: 20[11-40] | I: 12(32%) C:17(44%) p=0.277 | I: 4[3-5] C: 4[3-7] p=0.812 | 30day  I: 6(15%) C: 5(13%) |  |  |
| **Soares 2013** | 6MWD I:472.0[440.7-537.3] C:501.5[459.3-513.5] | 6MWD I:514.4[460.8-557.5] C:441.5[412.3-505.9] | 7 days after surgery  6MWD I: 368.5[272.3-408.5] C: 223.0[186.7-318.3]  30 days after surgery  6MWD I: 486.0 [392.3-562.3] C: 447.3[373.7-465.8] | | 2-3 | NR | I: 18[14-21] C: 14[14-21] | PPC I:5(31.3%) C: 11(78.6%) p=0.034 | I: 8.5[4.8-12.3] C:8.5[6.5-17.3] | N/A | I:1(5.6%) C:3(15.8%) |  |
|  | **Functional Measures** | | | |  |  |  | **Post-Operative Outcomes** | | | |  |
| **Study** | **Baseline** | **Pre-surgery** | **Post-op** | | **Prehab program designed (weeks)** | **Prehab delivered (days)** | **Time from baseline to surgery(days)** | **Complications, n(%)** | **Hospital Length of Stay, days** | **Readmission, n(%)** | **Morality, n(%)** |  |
| **CPET** |  |  |  | |  |  |  |  |  |  |  |  |
| **Blackwell 2020** | CPET AT (ml/kg/min) I:13.15±19 C: 13.84±2.8  VO2 Peak (ml/kg/min) I: 24.8±5.2 C:26.4±5.7 | CPET AT (ml/kg/min) Mean Diff from baseline 2.26ml/kg/min (95%CI 1.25 o 3.26)  VO2 Peak (ml/kg/min) Mean Diff from baseline 2.16ml/kg/min (95%CI 0.24 to 4.08) |  | | 4 | NR | NR | I:5(26%) C:3(14%) | - | I:1 (5%) C:0 |  |  |
| **Christensen 2019** | Peak VO2 (ml/min/kg) I: 25.23±8.38  30STS: I:13.9±3.6 C:12.7±3.9 | Peak VO2 (ml/min/kg)  I: 26.62±6.78 Mean difference +1.39 (0.03, 2.74) | N/A | | Baseline until surgery | 76.3±28.7 |  | All complications (CD≥II): I: 11[58], C: 13[57] RR 1.02 (95%CI 0.61-1.73)  Serious complications (C≥2): Total:9 I: 4, C: 5  CCI: I: 20.9[0-33.5] C:20.9[0-26.2] | I: 10[9-11] C:9[8-11] | - |  |  |
|  | **Functional Measures** | | | |  |  |  | **Post-Operative Outcomes** | | | |  |
| **Study** | **Baseline** | **Pre-surgery** | **Post-op** | | **Prehab program designed (weeks)** | **Prehab delivered (days)** | **Time from baseline to surgery(days)** | **Complications, n(%)** | **Hospital Length of Stay, days** | **Readmission, n(%)** | **Morality, n(%)** |  |
| **Banerjee 2018** | CPET: Peak VO2 I: 19.22±4.80 (17.32-21.12) C: 20.38±5.59 (18.07-22.68  AT I:11.49±2.08 (10.61–12.37) C: 11.38±2.57 (10.27–12.49) | CPET: Peak VO2  I: 21.07±5.60 (18.85-23.29) C: 20.84±5.43 (18.60-23.08)  AT I: 12.00±2.97 (10.74–13.25) C: 12.21±2.63 (11.07–13.35) | **N/A** | | 3-6 | NR | I: 32±6.5 (21-37) C: 29±4.2 (21-37) days  Baseline to repeat CPET | CD≥1 I: 4 (15%) C: 10 (36%) p=0.075  CD≥3 I: 1 (4%) C: 4 (14%) p=0.172 | I: median 7 (range 4-78) C: median 7 (range 5-107)  p=0.865 | - |  |  |
| **Dunne 2016** | CPET AT (ml/kg/min) I: 11.2±1.5 C:11.4±1.8  VO2 Peak (ml/kg/min) I:17.6±2.3 C:18.6±3.9 | CPET AT (ml/kg/min) I:12.2±2.4, p=0.093 C:11.0±2.1, p=0.088  VO2 Peak (ml/kg/min) I:19.6±3.8, p=0.019 C:18.7±4.1, p=0.958 | N/A | | 4 | NR | NR | Complications: all grades I:8 (42%) C:7 (47%)  CD (III-IV) I:3 (16%) C:1 (7%) | I: 5[4-6] C: 5[4.5-7] | I: 4 (21%) C: 0 (0%) |  |  |
| **Kaibori 2013** | CPET Not reported | CPET Not reported | CPET (1,3,6months) Standard (n=11)  AT VO2 at 6 mo (% of baseline): 102±14  High frequency (n=14)  Peak VO2 at 6 mo (% of baseline): 115±18 | | 4 | NR |  | Morbidity I: 2(9%) C: 3(13%) p=0.671 | I: 13.7±4.0 C: 17.5±11.3 p=0.120 |  | Mortality (yes/no) I: 0 C: 0 |  |
|  | **Functional Measures** | | | |  |  |  | **Post-Operative Outcomes** | | | |  |
| **Study** | **Baseline** | **Pre-surgery** | **Post-op** | | **Prehab program designed (weeks)** | **Prehab delivered (days)** | **Time from baseline to surgery(days)** | **Complications, n(%)** | **Hospital Length of Stay, days** | **Readmission, n(%)** | **Morality, n(%)** |  |
| **Dronkers 2010** | Physical Work Capacity (PWC), O_2_ ml/kg/min I: 29.4±9.5 C: 31.6±6.5  TUG(s)  I: 8.0±3.6 C:6.4±1.3  CRT I:26.3±6.7 C:21.6±4.7 | Physical Work Capacity (PWC), O_2_ ml/kg/min I: 27.6±6.5 C: 32.9±6.9  TUG(s)  I:7.8±3.3, p=0.29 C:6.6±1.2, p=0.28  CRT I:26.6±6.2, p=0.74 C:21.2±6.1, p=0.81 | N/A | | 2-4 | NR |  | Post-operative complications I: 9(45%) C:8(38%) p=0.65 | I:16.2±11.5 C:21.6±23.7 p=0.31 |  |  |  |
| **10MWT** |  |  |  | |  |  |  |  |  |  |  |  |
| **Ausania 2019** | 10mwt I: 6.03 C: Not reported | 10mwt I: 4.83 C: Not reported | N/A | | 1 | median 12.6 |  | I: 6 (33.3%) C: 12 (54.5%) p=0.18 Type III-IV I: 4 (22.2%) C: 4 (18.2%) p=0.751 | I:11.4 (7-46) C: 13.2 (7-60)  p=0.449 | I: 1(5.6%) C: 2(9.6%) p=0.673 |  |  |
|  | **Functional Measures** | | | |  |  |  | **Post-Operative Outcomes** | | | |  |
| **Study** | **Baseline** | **Pre-surgery** | **Post-op** | | **Prehab program designed (weeks)** | **Prehab delivered (days)** | **Time from baseline to surgery(days)** | **Complications, n(%)** | **Hospital Length of Stay, days** | **Readmission, n(%)** | **Morality, n(%)** |  |
| **Other** |  |  |  | |  |  |  |  |  |  |  |  |
| **Valkenet 2018** | N/A | N/A | N/A | | Baseline until surgery | 21(0-74) | 27(8-97) | Pneumonia: I: 47(39.2%) C: 43(35.5%)  RR 1.10(0.79,1.53) p=0.561  Antibiotics for suspected pneumonia: I:47(39.2%), C: 40(33.3%)  RR 1.119(0.84,1.66) p=0.326  Pulmonary, other: I: 41(34.7%) C: 40(33.3%) RR 1.04(0.73,1.49), p=0.818  Cardiac: I: 23(19.5%) C: 27(22.5%) RR 0.87(0.53,1.42) p=0.570  Complications, other: I: 26(22.0%) C:17(14.2%) RR 1.56(0.89,1.98) p=0.121 | I:18.4±18.0 C:20.5±20.9  RR 0.77(0.51,1.18), p=0.231  ICU I:3.3±7.5 C:3.1±6.6  RR 0.91(0.56,1.38) p=0.658 | ICU Readmission I: 12(10%)  C: 20(16.5%)  RR0.62(0.34, 1.17), p=0.138 | Mortality I:5(4.2%) C:3(2.5%)  RR1.67(0.40,6.87), p=0.478 |  |
|  | **Functional Measures** | | | |  |  |  | **Post-Operative Outcomes** | | | |  |
| **Study** | **Baseline** | **Pre-surgery** | **Post-op** | | **Prehab program designed (weeks)** | **Prehab delivered (days)** | **Time from baseline to surgery(days)** | **Complications, n(%)** | **Hospital Length of Stay, days** | **Readmission, n(%)** | **Morality, n(%)** |  |
| **Jensen 2015** | Nil | Nil | Nil | | 2 | NR |  | ≥1 complication I: 30(60%) C: 34(60%) p=0.47 | median(range) I: 8(3-30) C:8(4-55) p=0.68 | I:13(30) C:12(23) p=0.49 | Mortality within 90 days, n(%) I: 3(6) C:4(7) p=0.84 |  |
| **Yamana 2015** | N/A | N/A | N/A(4 days) | | Baseline until surgery | 15.1 |  | Complications, Clavien-Dindo score, n (%) I) I: 22 (73.3)  C: 12 (40), p=0.014 II) I: 5 (16.7) C: 13 (43.3) IIIa) I: 2 (6.7) C: 0  IIIb) I: 1 (3.3)  C: 5 (16.7) IV) I: 0 C: 0 V) I: 0 C: 0  Post op Pneumonia, Utrect Scoring System, n (%)  UPSS score POD1  0) I: 20 (66.7) C: 13 (43.3), p=0.031 1) I:10 (33.3) C: 12 (41.4)  2) I: 0 (0.0) C: 4 (13.8) 3) I: 0 (0.0) C: 1 (3.4)  UPSS score POD2  0) I: 8 (26.7) C: 11 (36.7), p= 0.87 1) I: 21 (70.0) C: 15 (50.0) 2) I: 1 (3.3) C: 3 (10.0) 3) I: 0 (0.0) C: 1 (3.3)  UPSS score POD3  0) I: 8 (26.7) C: 15 (50.0), p=0.13 1) I: 21 (70.0) C: 13 (43.3) 2) I: 1 (3.3) C: 1 (3.3) 3) I: 0 (0.0) C: 1 (3.3)  UPSS score POD4 (%)  0) I: 24 (80.0) C: 22 (73.3), p=0.51 1) I: 6 (20.0) C: 7 (23.3) 2) I: 0 (0.0) C: 0 (0.0) 3) I: 0 (0.0) C: 1 (3.3) |  |  |  |  |
| **Swaminathan 2020** | N/A | N/A | N/A | | 1 | NR | NR | I: 5(17%) C:14(48%) | I: 11[3] C:13[4] | NR |  |  |
| **Boden 2018** | N/A | N/A | N/A | | Single intervention anytime in the 6 weeks prior to surgery | 1 | N/A | PPC: I: 19(13%), C:43(29%)  Pneumonia: I:14(9.5%), C:33(22%)  Cardiac: I:9(6.1%), C:7(4.7%)  Wound infection: I:12(8.1%), C:15(10%)  Paralytic ileus: I:49(33%), C:67(45%) | ICU LoS: I:1.4±3.2, C:1.9±3.1  Acute LoS: I:11.0±11.7, C:12.2±11.2  Sub-acute LoS: I:0.9±4.3, C:1.0±5.3  Total hospital LoS: I:12.0±13.5, C:13.1±13.0 | 6-week hospital readmission:  I: 33(24%), C:29(21%) | 30 day mortality: I:3(2.0%), C:2(1.4%)  12 month mortality: I:15(10%), C:19(13%) |  |

Data are mean±SD(range), median[IQR].  * Number of completed intervention weeks. Abbreviations: I: Intervention, C: Control, LoS: length of stay, 6MWD: Six Minute Walk Distance, CPET End time: Cardiopulmonary Exercise Test Endurance Time, TUG, SCT; stair climb test, FTSTS: five times sit to stand, CD: Clavien-Dindo, 10mwt (meters): 10 meter walk test (sec). IQR: Interquartile range. OR: Odds ratio, HR: hazards ratio, CCI: Comprehensive Complications Index, 30STS: 30 second sit to stand test, CRT: Chair Rise Time (sec), STS: sit to stand test, N/A: not applicable, NR: not reported, NS: not statistically significant

# CERT Tables

## Table 2 – Description of Exercise Prehabilitation Intervention Arms According to Consensus Exercise Reporting Template (CERT) Domains Continued

| **CERT Domain** | **Item No.** | **Abbreviated Item Description** | **Bousquet-Dion 2018** | **Ausania 2019** |
| --- | --- | --- | --- | --- |
| What | 1 | Type of exercise equipment | Recumbent stepper, Resistance bands, Pedometer | Cycle-ergometer stationary bicycle |
| Who | 2 | Qualifications, teaching/supervising expertise and/or training of the exercise instructor | Kinesiologist | Physiotherapist |
| How | 3 | Whether exercise are performed individually or in a group | Not specified | Not specified |
|  | 4 | Whether exercises are supervised or unsupervised | Supervised and unsupervised home session | 5 days supervised, followed by unsupervised home-exercises |
|  | 5 | Measurement and reporting of adherence to exercise | Attendance at supervised sessions  Self-reported in diary and weekly telephone calls. | Patient reported exercise |
|  | 6 | Details of motivation strategies | Exercise instruction booklet, diary. Weekly telephone calls | Nil detailed |
|  | 7 | Decision rules for progressing the exercise program | Increased intensity with perceive mild exertion based on BORG (≤12/20) | Nil detailed |
|  | 8 | Each exercise is described so that it can be replicated (e.g., illustrations, photographs) | General description of muscle groups but not specific exercises. | Cycling program described in detail. No detail of home based functional training or breathing exercises |
|  | 9 | Content of any home program component | Aerobic and resistance training personalised to fitness level. Aerobic based on BORG and baseline 6MWT. Resistance based on 8 rep max. 3-4 days/week. Aerobic: 30min mod intensity (60-70%HRmax), walking/jogging/cycling. Resistance: 8 exercises, core, upper, lower limbs, x3-4/week, x2sets x8-15reps therabands. Pedometer for daily walks. | Functional exercises, but no detail  Breathing exercises. |
|  | 10 | Non exercised components | 60-min anxiety-reduction, relaxation and breathing exercise session, to repeat home 2-3/week.  Nutritional assessment+/- protein supplement | Nutritional support (oral supplement). Pancreatic enzyme replacement. Endocrine monitoring and treatment as needed. |
|  | 11 | How adverse events that occur during exercise are documented and managed | Not reported | Nil detailed |
| Where | 12 | Setting in which exercises are performed | Hospital exercise laboratory | Outpatient clinic |
| When, how much | 13 | Detailed description of the exercises (e.g. set, reps, ration, intensity) | Supervised:1xper week  W/U: 5 min Aerobic: 30 min mod intensity Resistance: 25 min, Stretching: 5 min | 5 sessions of 60 mins over 5 day period  Duration: 60min  W/U: 10 min  10 min muscle toning exercise 20 min aerobic exercise C/D: 10 min |
| Tailoring | 14 | Whether exercises are generic (“one size fits all”) or tailored to the individual | Personalised to HRmax and BORG | Personalised to each participant, no detail given |
|  | 15 | Decision rule that determines the starting level of exercise | Fitness level, BORG and 6MWT  Resistance: eight repetitions maximum test. | Multidisciplinary assessment, no specific detail |
| How well | 16 | Whether the exercise intervention is delivered and performed as planned | Compliance reported | Nil reported |

## Table 3 - Description of Exercise Prehabilitation Intervention Arms According to Consensus Exercise Reporting Template (CERT) Domains Continued

| **CERT Domain** | **Item No.** | **Christensen 2019** | **Dronkers 2010** | **Dunne 2016** |
| --- | --- | --- | --- | --- |
| What | 1 | Cycle ergomter and resistance gym equipment | Inspiratory muscle threshold loading device  Pedometer | Cycle ergometer |
| Who | 2 | Trained instructor | Physical therapist | Not stated for intervention. Testing by clinical psychologists |
| How | 3 | Not reported | Not specified | Not reported |
|  | 4 | Supervised | Supervised | Supervised |
|  | 5 | Attendance at hospital sessions Number of interruptions and exercise modifications | Attendance at scheduled visits to the supervised sessions | Attendance of supervised sessions |
|  | 6 | Not reported | Not reported | Not reported |
|  | 7 | Determined by trained instructor  HI: 85-95% HRmax, 1RM test | Aerobic and resistance: not specified, IMT: BORG scale 11-13 | Not reported |
|  | 8 | Detailed description in supplementary file with all specifics of programme, incl picture of equipment | Vague detail on exercises or mode of supervised aerobic. | Described intensity and number of sessions |
|  | 9 | Not reported | Walking or cycling: 30min per day.  Intensity: BORG 11-13  IMT (20% MIP x 15min/day). | Not reported |
|  | 10 | None reported | None reported | Not reported |
|  | 11 | Well documented – incl dose reduction or early termination as well as attendance (suppl file) | No adverse events reported | No adverse events occurred |
| Where | 12 | Hospital PA research unit | Hospital outpatient department | Exercise laboratory |
| When, how much | 13 | 2xp/wk 75min session  W/U: 10 min cycling  HIIT: 21-28mins - 4x4min with 3min active recovery  Resistance: 1xW/U set, 3sets x8-12reps. 4 exercises; chest press, leg press, lateral pull and knee extension. | 2-4weeks 60mins duration W/U, resistance leg extensors (1 set x8-15reps= 60-80% 1RM).  IMT x15min  Aerobic: 20-30min (55-75% HRmax) or BORG 11-13  Functional activities,C/D | 12 sessions over 4 week period  W/U  Interval training:30min alternating between moderate (<60%VO2peak) and vigorous (>90% VO2peak), C/D |
| Tailoring | 14 | Personalised to individual fitness and strength | Tailored to HRmax, BORG, 1RM, MIP | Intensity tailored |
|  | 15 | HI: 85-95% HRmax  1RM test | HRmax, BORG, 1RM, MIP | Standardized equation based on work rate at AT of baseline CPET |
| How well | 16 | Detailed adherence data with session adherence and modifications. | Reported high attendance to supervised sessions, patient appreciation of treatment questionnaire. | Attendance of sessions |

## Table 4 - Description of Exercise Prehabilitation Intervention Arms According to Consensus Exercise Reporting Template (CERT) Domains Continued

| **CERT Domain** | **Item No.** | **Gillis 2014** | **Jensen 2015** | **Kaibori 2013** |
| --- | --- | --- | --- | --- |
| What (materials) | 1 | Resistance bands x3 | Step trainer | Nil |
| Who (provider) | 2 | Kinesiologist | Physiotherapists | Medical doctor and exercise trainer |
| How (delivery) | 3 | Individual | Individual | Not reported |
|  | 4 | Unsupervised | Unsupervised | Not reported |
|  | 5 | Weekly telephone calls, Patient diary | Self-reported in diary | Not reported pre-op |
|  | 6 | Instructional booklet, Weekly telephone calls | Discussion of mutual expectations and motivation. Telephone call after 1 week. | Not reported |
|  | 7 | BORG and/or reps x15 | Patients were encouraged to progress through training programme by increasing number of reps (no detail on when) | Post-op review by doctor and trainer |
|  | 8 | Aerobic exercise described, Resistance exercises generic description | No description or pictures provided | Not reported |
|  | 9 | All home-based | All home based | None reported |
|  | 10 | Nutrition and  psychological support | Information/education lifestyle. | Diet input |
|  | 11 | Not reported | Not reported | Not reported |
| Where (location) | 12 | Home based | Home based | Not reported |
| When, how much (dosage) | 13 | 8 weeks, 3 days per week, 50 min per session, alternating resistance and aerobic ex.  Aerobic – type at pt discretion, W/U 5 mins, 20min aerobic ex starting at 40%HRR, 20min resistance (8 exercise major muscle groups at 8-12reps max, C/D 5min) | 2 weeks pre-op 2xday  Aerobic – 15min  Resistance – six different muscle strength and endurance exercises | 3x60min sessions p/wk,  5min stretching,  30min walking,  20min targeted stretching,  5min C/D with stretching |
| Tailoring (what, how) | 14 | HR, BORG | Individualised reps | Tailored |
|  | 15 | BORG from 6MWT and %HRmax (Karvonen) | Not reported | Intensity based on AT during CPET |
| How well (planned, actual) | 16 | Self-reported compliance | Compliance to program recorded. | Analysis split for standard 3/week exercisers vs high frequency 5-6/week – confirmed by review by doctor and trainer |

## Table 5 - Description of Exercise Prehabilitation Intervention Arms According to Consensus Exercise Reporting Template (CERT) Domains Continued

| **CERT Domain** | **Item No.** | **Karlsson 2019** | **Minnella 2019** | **Banerjee 2018** |
| --- | --- | --- | --- | --- |
| What (materials) | 1 | Power Breathe K3, Weighted belts | Resistance bands | Cycle-ergometer |
| Who (provider) | 2 | Physiotherapist | Kinesiologist | Exercise science staff |
| How (delivery) | 3 | Individual | Individual | Not specified |
|  | 4 | Supervised | Unsupervised, after intro session with kinesiologist | Supervised |
|  | 5 | IMT – record through device. Exercise diary | weekly telephone calls to record adherence Patient logbooks | Attendance at supervised sessions |
|  | 6 | Not stated | Weekly telephone calls | Nil detailed |
|  | 7 | IMT – single breath test, increased 5% based on BORG  Strength – chair stand test and BORG | Not reported | Increased load to maintain target BORG/%HR max |
|  | 8 | Detailed description | Detailed description | Detailed description provided. |
|  | 9 | All home based  Unsupervised advised to follow 150min/wk recommendations and complete core functional ex 2-3/week and IMT x30 breaths/day | All home based | Nil detailed |
|  | 10 | Nil | Nutrition assessment and supplements as needed.  Anxiety-reduction. | Nil detailed |
|  | 11 | Recorded by PT in logs, by patients in diaries | No adverse events occurred | No adverse events |
| Where (location) | 12 | Home based | Home based | University exercise facility |
| When, how much (dosage) | 13 | 1hr sessions 2-3/wk for minimum 2 wks IMT @50% max capacity x30 breaths x2/day  High intensity functional strength exercises 3x10reps BORG 7-8.  Endur training – intervals | 3xper week  Aerobic (walking, jogging, cycling) 5min W/U, x25min mod intensity continuous exercise (RPE 12-13/20 BORG)  10min C/D and stretching  Strengthening: 25min mod-intensity 3sets x8-12 reps 8 muscle groups | Twice weekly W/U: 5-10min, 50W Intervals: 6x5min @BORG 13-15, 70-85% predicted HRmax  Active Rest: 2.5min 50W  Cadence 50-60 RPM  C/D: 50 W |
| Tailoring (what, how) | 14 | Tailored, use of Patient Specific Functional Scale | Personalised on fitness, preference, available resources | Tailored HRmax and BORG |
|  | 15 | MIP, BORG on chair stand test and step up with weight belt | BORG RPE | BORG scale |
| How well (planned, actual) | 16 | Reported high compliance with programme 97%. Good reporting of dose and intensity of programme | Reported high adherence to protocol median 83.3% | Based on predicted HRmax and HR peak. Number of intervals achieved reported. |

## Table 6 - Description of Exercise Prehabilitation Intervention Arms According to Consensus Exercise Reporting Template (CERT) Domains Continued

| **CERT Domain** | **Item No.** | **Moug 2019** | **Northgraves 2019** | **Valkenet 2018** |
| --- | --- | --- | --- | --- |
| What (materials) | 1 | Pedometer | Cycle ergometer, resistance bands, hand weights | Inspiratory loading device (Powerbreathe) |
| Who (provider) | 2 | study coordinator with behaviour change theory training | Certified strength and conditioning instructor | Physiotherapist |
| How (delivery) | 3 | Individual | Individual | Individual |
|  | 4 | Unsupervised | Supervised | 1 face to face supervised session, followed by unsupervised home sessions |
|  | 5 | Self-report diary,  Number of telephone calls planned and received by participants. | Logged by instructor  Self-reported diary of PA | Self-report in diary  Weekly telephone calls with physio interviews |
|  | 6 | Behaviour change theory – self-regulatory and health action process approach.  Diary with targets and motivational material. Asked to engage supportive person to assist (spouse). | Motivation, and encouragement was provided throughout each session | Diary, instructional video, follow up telephone call |
|  | 7 | Based on baseline step count – graduated goals aiming to increase avg step count by 3000/day | Progressions every 2-3sessions dependent on individual ability, increased reps or resistance or duration.  Aerobic – increased 2-5mins per session up to 25min | Increase 5% if RPE<7 |
|  | 8 | Walking programme example outlined. | Detailed description of dose/intensity in supplemental info. All exercises described specifically. | General description of procedure |
|  | 9 | All home based | None detailed | Predominantly home based |
|  | 10 | Motivational techniques | None detailed | None detailed |
|  | 11 | No adverse events occurred | One unrelated adverse event (in supplemental table) | No serious adverse events were reported during the trial. |
| Where (location) | 12 | Home based/Community | University Sport Science Laboratory | Outpatient setting and home |
| When, how much (dosage) | 13 | Increased step count  Wk1-2 x1500steps x3days  Wk3-4 x1500 x5days  Wk5-6 x3000 x3days  Wk7-8 x3000 x5days  Wk9-17 maintain or increase step count | 60min 3xp/wk  5min W/U @40-50%HRR  Resistance (functional training) circuit 3-4sets  Aerobic – moderate intensity up to 25min cycling @40-60%HRR and/or BORG 11-13  Resistance Circuit x3-4sets, C/D x5min | x30 breaths x2/day, 7 days/week, for ≥2 weeks |
| Tailoring | 14 | Generic | Tailored | Tailored based on RPE |
|  | 15 | Baseline step count from accelerometer | CPET calculated HRR | Starting at 60% baseline MIP |
| How well (planned, actual) | 16 | High adherence based on performed calls and self-reported sessions | High attendance (89.6% range 75-100%). Recorded detail of reasons for nonattendance. | Training adherence and compliance reported in supplementary information |

## Table 7 - Description of Exercise Prehabilitation Intervention Arms According to Consensus Exercise Reporting Template (CERT) Domains Continued

| **CERT Domain** | **Item No.** | **Yamana 2015** | **Swaminathan 2020** | **Soares 2013** |
| --- | --- | --- | --- | --- |
| What (materials) | 1 | Cycle ergometer, weights | Incentive Spirometry (Coach 2® volume-oriented incentive spirometer, Smiths Medical International Ltd., USA) | Inspiratory threshold-loading device, (Threshold IMT, Respironics, NJ, USA) |
| Who (provider) | 2 | Physical therapist | Not reported | Not stated |
| How (delivery) | 3 | Individual | Individually | Not stated |
|  | 4 | Supervision | Unsupervised | Supervised and unsupervised |
|  | 5 | Not reported | Patient asked to keep a daily diary, noting incentive spirometry volume generated | Not reported |
|  | 6 | None detailed | Not reported | Not reported |
|  | 7 | Not reported | Not reported | IMT: increased by 2cmH2O/week in the preoperative period. |
|  | 8 | Respiratory activities/exercises listed with some detail. Strength exercises for legs and abdominals not detailed. Cycling load/intensity not reported | Not reported | General exercise type described (e.g. upper extremity) but no specific details or figures |
|  | 9 | None detailed | Incentive spirometry use every 4 hours, 15 times per session over a period of 7 days, preoperatively | 4x/wk: respiratory muscle training (15mins) and walking (10mins) were carried out once daily on non-supervised exercise days |
|  | 10 | Reported no adverse events | Not applicable | Coughing and huffing education |
|  | 11 | Intensive PR was well-tolerated by all patients with no adverse events. | Not reported | Not reported |
| Where (location) | 12 | Rehabilitation centre during hospital stay | Home based | Participants recruited in private university and a tertiary public hospital however unclear where exercise was performed. |
| When, how much (dosage) | 13 | Whole programme 60min per day x >7 days, no specific detail on reps/intensity  Cycling x20min duration | Incentive spirometry use every 4 hours, 15 times per session over a period of 7 days | 2x 50 minute supervised sessions/wk: stretching exercises, trunk rotation, deep breathing, respiratory muscle training(15min), active upper and lower extremity exercises, walking (10 min) on flat ground and relaxation. |
| Tailoring (what, how) | 14 | Individual tailoring to patient’s background | Generic | Generic program. IMT load and walking intensity tailored to the participants. Walking <15 on BORG RPE scale. |
|  | 15 | No detail reported | Method to determine starting level not reported. Level of practice consistent across all participants | IMT initial load was set at 20% of their maximal inspiratory pressure, measured at baseline. |
| How well (planned, actual) | 16 | Not reported | Not reported | Preoperative data was collected for all subjects however adherence to supervised exercise program components or deviations from the protocol were not stated. 100% adherence reported by all participants to all preoperative unsupervised components. |

## Table 8 - Description of Exercise Prehabilitation Intervention Arms According to Consensus Exercise Reporting Template (CERT) Domains Continued

| **CERT Domain** | **Item No.** | **Blackwell 2020** | **Boden 2018** |
| --- | --- | --- | --- |
| What (materials) | 1 | Cycle ergometer | None |
| Who (provider) | 2 | Medical doctor | Physiotherapists with experience ranging from students to 15 years acute surgical practice and education. All watched a training video and were given a semi-structured education session guide. |
| How (delivery) | 3 | Individually | Not reported |
|  | 4 | Supervised | Supervised |
|  | 5 | Attendance at supervised sessions | Not applicable |
|  | 6 | Not reported | Not applicable |
|  | 7 | Increase in wattage implemented mid-way point of training to maintain exercise intensity | Not applicable |
|  | 8 | Description | Not included |
|  | 9 | Participants encouraged to maintain habitual physical activity and dietary regimes | Not applicable |
|  | 10 | No non-exercise components | Education and memory cues |
|  | 11 | No adverse events occurred during training | No adverse events occurred during the intervention |
| Where (location) | 12 | University exercise laboratory | Hospital pre-admission clinic |
| When, how much (dosage) | 13 | 12 HIIT sessions (3-4xp/wk, with no training on weekends) within a 4-week period (<31 days  W/U: 2 mins of unloaded cycling  Main set: 5x1min exertions at 100-115% of the maximal load (watts(W)) reached during initial CPET, ending with a 2 min recovery period of unloaded cycling. | 2 sets of 10 slow deep breaths followed by three coughs to be performed hourly and starting immediately after surgery |
| Tailoring (what, how) | 14 | Intensity tailored to the individual but not exercise type. | Generic |
|  | 15 | Starting level based on 100-115% of initial CPET maximum wattage | Generic |
| How well (planned, actual) | 16 | Attendance at supervised sessions (adherent participants defined as those who attended ten or more sessions - 84%). | Single intervention delivered to all participants; adherence post-operatively not reported |

**Table 9: Use of the GRADE[6] approach for each meta-analysis of cardiorespiratory fitness or functional capacity to establish quality of evidence.**

| **Outcome**  No of studies  No. of participants | **Study design grade** | **Risk of bias / quality**  (Downgrade 1 if ≥50% of domains rated as ‘some concerns’ or ‘high’ on risk of bias on Cochrane risk-of-bias tool version 2 excluding ‘Selection of the reported result’*) | **Heterogeneity or inconsistency of effect**  (Potential downgrade if wide variance of point estimates across studies; minimal/no overlap of CIs; wide (>2) PI or significant heterogeneity tests) | **Indirectness of evidence**  (Downgrade 1 if significant differences in PICO factors that may result in change of outcome) | **Imprecision**  (Does the 95% CI cross the line of no effect? If yes, Downgrade 1.  If no, Is the OIS† reached? If no, Downgrade 1 | **Publication bias**‡ | **Final Grade** |
| --- | --- | --- | --- | --- | --- | --- | --- |
| **VO_2_ peak[7-9]**  3 RCTs  n=121 | High | Low risk (93% 14/15 domains)  = No change | Wide PI interval (-9.67 to 13.15) = Downgrade 1 | No important indirectness = no change | 95%CI does not exclude 0 = Downgrade 1 | Only 3 small trials, point estimates all positive however publication bias unlikely to be affected as primary outcome in 2/3 included studies was AT = No change | Low |
| **AT[7-9]**  3 RCTS  n=121 | High | Low risk (93% 14/15 domains)  = No change | Important inconsistency and wide PI (-16.33 to 18.75) = Downgrade 1 | No important indirectness = no change | 95%CI does not exclude 0 = Downgrade 1 | Point estimates not all positive, only 3 small trials = No change | Low |
| **6MWT[10-17]**  8 RCTs  n=437 | High | Low risk (91% low risk 32/35 domains)  = No change | No important inconsistency = No change | Differences in timing of outcomes = Downgrade 1 | 95%CI excludes 0. OIS met as per power calculation in Gillis et al. 2014 = No change | Point estimates all positive, however study size sufficient = No change | Moderate |

Abbreviations: GRADE: Grades of Research, Assessment, Development and Evaluation, VO_2_ peak: Peak oxygen consumption, AT: Anaerobic Threshold, 6MWT: Six Minute Walk Test, CI: Confidence Interval, PI: Prediction interval. *Risk of bias was determined using the Cochrane risk-of-bias tool version 2 excluding ‘Selection of the reported results’ domain. It was felt by the authors that this domain skewed the overall risk of bias score, given it was scored ‘some concerns’ if a trial did not have a pre-specified statistical analysis plan prior to unblinding of outcome data and reporting of this may not have been common practice until more recently. †Where there was no power calculation for the outcome, the OIS required was presumed to be met if n≥400 [6]. ‡Publication bias decision was based around the following considerations: study design, study size and symmetry of the funnel plot if ≥10 trials included in the meta-analysis [6].

**Table 10: Use of the GRADE[6] approach for each meta-analysis of post-operative outcomes to establish quality of evidence.**

| **Outcome**  No of studies  No. of participants | **Study design grade** | **Risk of bias / quality**  (Downgrade 1 if ≥50% of domains rated as ‘some concerns’ or ‘high’ on risk of bias on Cochrane risk-of-bias tool version 2) | **Heterogeneity or inconsistency of effect**  (Potential downgrade if wide variance of point estimates across studies; minimal/no overlap of CIs; wide (<2) PI or significant heterogeneity tests) | **Indirectness of evidence**  (Downgrade 1 if significant differences in PICO factors that may result in change of outcome) | **Imprecision**  (Does the 95% CI cross the line of no effect? If yes, Downgrade 1.  If no, Is the OIS† reached? If no, Downgrade 1) | **Publication bias**‡ | **Final Grade** |
| --- | --- | --- | --- | --- | --- | --- | --- |
| **Postoperative Complications**  16 RCTs[7-15, 17-23]  n=917 | High | Low risk (83% 67/80 domains)  = No change | No important inconsistency = No change | Differences in outcome measures used to assess outcome = Downgrade 1 | 95%CI does not exclude 0 = Downgrade 1 | Variability in point estimates (including 2 null and 2 negative) = No change | Low |
| **Postoperative Pulmonary Complications**  7 RCTs[9, 12, 14, 20, 24-26]  n=845 | High | Low risk (83% 29/35 domains)  = No change | No important inconsistency = No change | Difference in outcome measures used to assess outcome = Downgrade 1 | 95%CI does not exclude 0 = Downgrade 1 | Variability in results (2 trials with negative point estimates) = No change | Low |
| **Hospital LoS**  4 RCTs[12, 20, 24, 27]  n=458 | High | Low risk (80% 16/20 domains)  = No change | Important inconsistency with wide PI noted = Downgrade 1 | No important indirectness = No change | 95%CI excludes 0. Unclear whether OIS was met however sample size likely allows for balance of prognostic factors between groups = No change | Only 4 trials, all positive point estimates however study size appropriate = No change | Moderate |
| **Hospital Re-admission**  6 RCTs[13-17, 19]  n=477 | High | Low risk (90% 27/30 domains)  = No change | No important inconsistency = No change | No important indirectness = No change | 95%CI does not exclude 0 = Downgrade 1 | Variability in results (4 trials with null or negative point estimates) = No change | Moderate |
| **Postoperative Mortality**  7 RCTs[12, 15, 19, 24-27]  n=903 | High | Low risk (80% 28/35 domains)  = No change | No important inconsistency = No change | Difference in timepoints collected = Downgrade 1 | 95%CI does not exclude 0 = Downgrade 1 | Variability in results (3 trials with negative point estimates) = No change | Low |

Abbreviations: GRADE: Grades of Research, Assessment, Development and Evaluation, LoS: Length of stay, CI: Confidence Interval, PI: Prediction interval.

*Risk of bias was determined using the Cochrane risk-of-bias tool version 2 excluding ‘Selection of the reported results’ domain. It was felt by the authors that this domain skewed the overall risk of bias score, given it was scored ‘some concerns’ if a trial did not have a pre-specified statistical analysis plan prior to unblinding of outcome data and reporting of this may not have been common practice until more recently. †Where there was no power calculation for the outcome, the OIS required was presumed to be met if n≥400 [6]. ‡Publication bias decision was based around the following considerations: study design, study size and symmetry of the funnel plot if ≥10 trials included in the meta-analysis [6].

Postoperative Complications Funnel Plot


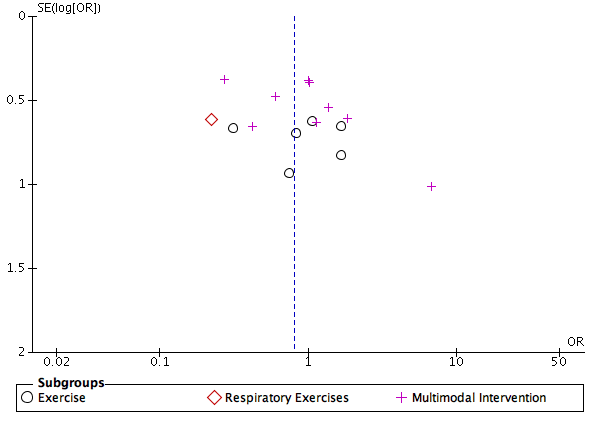


# Table 11 – Pooled analysis of data included in meta-analyses

| **Outcome** | **Trials (n)** | **Participants (n)** | **MD (95% CI); p value 95% PI** |
| --- | --- | --- | --- |
| **VO2 Peak** | 3 | 121 | 1.74 (-0.03 to 3.50); 0.05  -9.67 to 13.15 |
| **AT** | 3 | 121 | 1.21 (-0.34 to 2.76); 0.13  -16.33 to 18.75 |
| **6MWD** | 8 | 522 | 34.11 (19.13 to 49.08); <0.01  15.42 to 52.80 |
| **6MWD**  *After Multimodal Prehabilitation* | 6 | 464 | 33.09 (17.69 to 48.50); <0.01  11.26 to 54.92 |
| **Hospital LOS** | 4 | 458 | -3.68 (-6.44 to -0.92); 0.01  -9.74 to 2.38 |
| **Outcome** | **Trials (n)** | **Participants (n)** | **Odds Ratio (95% CI); p value 95% PI** |
| **Complications** | 16 | 917 | 0.81 (0.55 to 1.18); 0.27  0.26 to 2.50 |
| **Complications**  *Pulmonary* | 7 | 845 | 0.53 (0.28 to 1.01); 0.05  0.09 to 3.02 |
| **Hospital Readmission** | 6 | 464 | 1.07 (0.61 to 1.90); 0.81  0.47 to 2.41 |
| **Mortality** | 7 | 901 | 0.95 (0.43 to 2.09); 0.90  0.34 to 2.67 |

Abbreviations: 6MWD: six minute walk distance, VO2 Peak: peak oxygen consumption, LOS: length of stay, MD: mean difference, CI: confidence interval, PI: prediction interval, OR: odds ratio

# References

1. Slade, S., et al., *CERT Delphi panel. Consensus on Exercise Reporting Template (CERT): modified Delphi study. .* RPhys Ther., 2016. **96**: p. 1514–1524.

2. Hoffmann, T.C., C. Erueti, and P.P. Glasziou, *Poor description of non-pharmacological interventions: analysis of consecutive sample of randomised trials.* BMJ (Clinical research ed.), 2013. **347**: p. f3755-f3755.

3. Gianola, S., et al., *Reporting of Rehabilitation Intervention for Low Back Pain in Randomized Controlled Trials: Is the Treatment Fully Replicable?* Spine (Phila Pa 1976), 2016. **41**(5): p. 412-8.

4. Page, P., B. Hoogenboom, and M. Voight, *IMPROVING THE REPORTING OF THERAPEUTIC EXERCISE INTERVENTIONS IN REHABILITATION RESEARCH.* International journal of sports physical therapy, 2017. **12**(2): p. 297-304.

5. Borenstein, M., *Research Note: In a meta-analysis, the I2 index does not tell us how much the effect size varies across studies.* Journal of Physiotherapy (Elsevier), 2020. **66**(2): p. 135-139.

6. Schünemann, H., et al., *GRADE handbook for grading quality of evidence and strength of recommendations. Updated October 2013. The GRADE Working Group, 2013*.

7. Banerjee, S., et al., *Vigorous intensity aerobic interval exercise in bladder cancer patients prior to radical cystectomy: a feasibility randomised controlled trial.* Supportive Care in Cancer, 2018. **26**(5): p. 1515-1523.

8. Blackwell, J.E.M., et al., *High-intensity interval training produces a significant improvement in fitness in less than 31 days before surgery for urological cancer: a randomised control trial.* Prostate Cancer Prostatic Dis, 2020.

9. Dunne, D.F., et al., *Randomized clinical trial of prehabilitation before planned liver resection.* British Journal of Surgery, 2016. **103**(5): p. 504-512.

10. Moug, S.J., et al., *Prehabilitation is feasible in patients with rectal cancer undergoing neoadjuvant chemoradiotherapy and may minimize physical deterioration: results from the REx trial.* Colorectal Disease 2019 May;21(5):548-562, 2019.

11. Northgraves, M.J., et al., *Feasibility of a novel exercise prehabilitation programme in patients scheduled for elective colorectal surgery: a feasibility randomised controlled trial.* Supportive Care in Cancer, 2019.

12. Barberan-Garcia, A., et al., *Personalised prehabilitation in high-risk patients undergoing elective major abdominal surgery: a randomized blinded controlled trial.* Annals of Surgery 2018 Jan;267(1):50-56, 2018.

13. Bousquet-Dion, G., et al., *Evaluation of supervised multimodal prehabilitation programme in cancer patients undergoing colorectal resection: a randomized control trial.* Acta Oncologica, 2018. **57**(6): p. 849-859.

14. Gillis, C., et al., *Prehabilitation versus Rehabilitation: A Randomized Control Trial in Patients Undergoing Colorectal Resection for Cancer.* Anesthesiology, 2014. **121**(5): p. 937-947.

15. Minnella, E.M. and F. Carli, *Prehabilitation and functional recovery for colorectal cancer patients.* European Journal of Surgical Oncology, 2018. **44**(7): p. 919-926.

16. Minnella, E.M., et al., *Multimodal Prehabilitation to Enhance Functional Capacity Following Radical Cystectomy: a Randomized Controlled Trial.* European Urology Focus, 2019.

17. Carli, F., et al., *Effect of Multimodal Prehabilitation vs Postoperative Rehabilitation on 30-Day Postoperative Complications for Frail Patients Undergoing Resection of Colorectal Cancer: A Randomized Clinical Trial.* JAMA Surgery, 2020. **22**: p. 22.

18. Ausania, F., et al., *Prehabilitation in patients undergoing pancreaticoduodenectomy: a randomized controlled trial.* Revista Espanola de Enfermedades Digestivas, 2019. **111**.

19. Jensen, B.T., et al., *Efficacy of a multiprofessional rehabilitation programme in radical cystectomy pathways: a prospective randomized controlled trial.* Scandinavian Journal of Urology, 2015. **49**(2): p. 133-141.

20. Dronkers, J.J., et al., *Preoperative therapeutic programme for elderly patients scheduled for elective abdominal oncological surgery: a randomized controlled pilot study.* Clinical Rehabilitation, 2010. **24**(7): p. 614-622.

21. Swaminathan, N., et al., *ERAS protocol with respiratory prehabilitation versus conventional perioperative protocol in elective gastrectomy- a randomized controlled trial.* Int J Surg, 2020.

22. Christensen, J.F., et al., *Safety and feasibility of preoperative exercise training during neoadjuvant treatment before surgery for adenocarcinoma of the gastro-oesophageal junction.* Bjs Open, 2019. **3**(1): p. 74-84.

23. Karlsson, E., et al., *Feasibility of preoperative supervised home-based exercise in older adults undergoing colorectal cancer surgery -- a randomized controlled design.* PLoS ONE 2019 Jul;14(7):e0219158, 2019.

24. Valkenet, K., et al., *Multicentre randomized clinical trial of inspiratory muscle training versus usual care before surgery for oesophageal cancer.* British Journal of Surgery, 2018. **105**(5): p. 502-511.

25. Boden, I., et al., *Preoperative physiotherapy for the prevention of respiratory complications after upper abdominal surgery: pragmatic, double blinded, multicentre randomised controlled trial.* BMJ, 2018. **360**(j5916).

26. Soares, S.M., et al., *Pulmonary function and physical performance outcomes with preoperative physical therapy in upper abdominal surgery: a randomized controlled trial.* Clin Rehabil, 2013. **27**(7): p. 616-27.

27. Kaibori, M., et al., *Perioperative exercise for chronic liver injury patients with hepatocellular carcinoma undergoing hepatectomy.* American Journal of Surgery, 2013. **206**(2): p. 202-209.
